# Towards Improved Molecular Identification Tools in Fine Fescue (*Festuca* L., Poaceae) Turfgrasses: Nuclear Genome Size, Ploidy, and Chloroplast Genome Sequencing

**DOI:** 10.3389/fgene.2019.01223

**Published:** 2019-12-06

**Authors:** Yinjie Qiu, Cory D. Hirsch, Ya Yang, Eric Watkins

**Affiliations:** ^1^ Department of Horticultural Science, University of Minnesota, St. Paul, MN, United States; ^2^ Department of Plant Pathology, University of Minnesota, St. Paul, MN, United States; ^3^ Department of Plant and Microbial Biology, University of Minnesota, St. Paul, MN, United States

**Keywords:** fine fescue, chloroplast genome, phylogeny, comparative genomics, low input turfgrass

## Abstract

Fine fescues (*Festuca* L., Poaceae) are turfgrass species that perform well in low-input environments. Based on morphological characteristics, the most commonly-utilized fine fescues are divided into five taxa: three are subspecies within *F. rubra* L. and the remaining two are treated as species within the *F. ovina* L. complex. Morphologically, these five taxa are very similar; both identification and classification of fine fescues remain challenging. In an effort to develop identification methods for fescues, we used flow cytometry to estimate genome size and ploidy level and sequenced the chloroplast genome of all five taxa. Fine fescue chloroplast genome sizes ranged from 133,331 to 133,841 bp and contained 113–114 genes. Phylogenetic relationship reconstruction using whole chloroplast genome sequences agreed with previous work based on morphology. Comparative genomics suggested unique repeat signatures for each fine fescue taxon that could potentially be used for marker development for taxon identification.

## Introduction

With ca. 450 species, Fescues (*Festuca* L., Poaceae) is a large and diverse genus of perennial grasses (Clayton and Renvoize, 1986). Fescue species are distributed mostly in temperate zones of both the northern and southern hemispheres, but most commonly found in the northern hemisphere (Jenkin, 1959). Several of the fescue species have been commonly used as turfgrass. Based on both leaf morphology and nuclear ITS sequences, fescue species can be divided into two groups: broad-leaved fescues and fine-leaved fescues (Torrecilla and Catalán, 2002). Broad-leaved fescues commonly used as turfgrass include tall fescue (*Festuca arundinacea* Schreb.) and meadow fescue (*Festuca pratensis* Huds.). Fine-leaved fescues are a group of cool-season grasses that include five commonly used taxa called fine fescues. Fine fescues include hard fescue (*Festuca brevipila* Tracey, 2n = 6x = 42), sheep fescue (*Festuca ovina* L., 2n = 4x = 28), strong creeping red fescue (*Festuca rubra* ssp. *rubra* 2n = 8x = 56), slender creeping red fescue [*F. rubra* ssp. *litoralis* (G. Mey.) Auquier 2n = 6x = 42], and Chewings fescue [*F. rubra* ssp. *fallax* (Thuill.) Nyman 2n = 6x = 42] (Ruemmele et al., 1995). All five taxa share very fine and narrow leaves and have been used for forage, turf, and ornamental purposes. They are highly tolerant to shade and drought and prefer low pH (5.5-6.5) and low fertility soils (Beard, 1972). Additionally, fine fescues grow well in the shade or sun, have reduced mowing requirements, and do not need additional fertilizer or supplemental irrigation (Ruemmele et al., 1995).

Based on morphological and cytological features, fine fescues are currently divided into two groups referred to as the *F. rubra* complex (includes *F. rubra* ssp. *litoralis*, *F. rubra* ssp. *rubra*, and *F. rubra* ssp. *fallax*) and the non-rhizomatous *F. ovina* complex (includes *F. brevipila* and *F. ovina*) (Ruemmele et al., 1995). While it is relatively easy to separate fine fescue taxa into their proper complex based on the presence and absence of rhizome, it is challenging to identify taxon within the same complex. In the *F. rubra* complex, both ssp. *litoralis* and ssp. *rubra* are rhizomatous, while ssp. *fallax* is non-rhizomatous. However, the separation of ssp. *litoralis* from ssp. *rubra* using rhizome length is challenging. Taxon identification within the *F. ovina* complex relies heavily on leaf characters; however, abundant morphological and ecotype diversity within *F. ovina* makes taxa identification difficult (Piper, 1906). This is further complicated by inconsistent identification methods between different continents. For example, in the United States, sheep fescue is described as having a bluish gray leaf color and hard fescue leaf blade color is considered green (Beard, 1972), while in Europe, it is the opposite (Hubbard, 1968). Because the ploidy level of the five taxa varies from tetraploid to octoploid, beyond morphological classifications, laser flow cytometry has been used to determine ploidy level of fine fescues and some other fescue species (Huff and Palazzo, 1998). A wide range of DNA contents within each complex suggests that the evolutionary history of each named species is complicated, and interspecific hybridization might interfere with species determination using this approach. Plant breeders have been working to improve fine fescues for turf use for several decades, with germplasm improvement efforts focused on disease resistance, traffic tolerance, and ability to perform well under heat stress (Casler, 2003). Turfgrass breeders have utilized germplasm collections from old turf areas as a source of germplasm (Bonos and Huff, 2013); however, confirming the taxon identity in these collections has been challenging. A combination of molecular markers and flow cytometry could be a valuable tool for breeders to identify fine fescue germplasm (Hebert et al., 2003).

Due to the complex polyploidy history of fine fescues, sequencing plastid genomes provides a more cost-effective tool for taxon identification than the nuclear genome because it is often maternally inherited, lacks heterozygosity, is present in high copies, and is usable even in partially degraded material (Bryan et al., 1999; Provan et al., 2001). Previous studies have developed universal polymerase chain reaction (PCR) primers to amplify non-coding polymorphic regions for DNA barcoding in plants for species identification (Baldwin et al., 1995; Demesure et al., 1995). However, the polymorphisms discovered from these regions are often single nucleotide polymorphisms that are difficult to apply using PCR screening methods. For these reasons, it would be helpful to assemble chloroplast genomes and identify simple sequence repeat (SSR) and tandem repeats polymorphisms. Chloroplast genome sequencing has been simplified due to improved sequencing technology. In turfgrass species, high throughput sequencing has been used to assemble the chloroplast genomes of perennial ryegrass (*Lolium perenne* cv. Cashel) (Diekmann et al., 2009), tall fescue (*Lolium arundinacea* cv. Schreb) (Cahoon et al., 2010), diploid *F. ovina*, *F. pratensis*, *Festuca altissima* (Hand et al., 2013), and bermudagrass (*Cynodon dactylon*) (Huang et al., 2017). To date, there is limited molecular biology information on fine fescue taxon identification and their phylogenetic position among other turfgrass species (Hand et al., 2013; Cheng et al., 2016). In this study, we used flow cytometry to confirm the ploidy level of five fine fescue cultivars, each representing one of the five commonly utilized fine fescue taxa. We then reported the complete chloroplast genome sequences of these five taxa and carried out comparative genomics and phylogenetic inference. Based on the genome sequence we identified unique genome features among fine fescue taxa and predicted taxon specific SSR and tandem repeat loci for molecular marker development.

## Results

### Species Ploidy Level Confirmation

**Figure 1:**
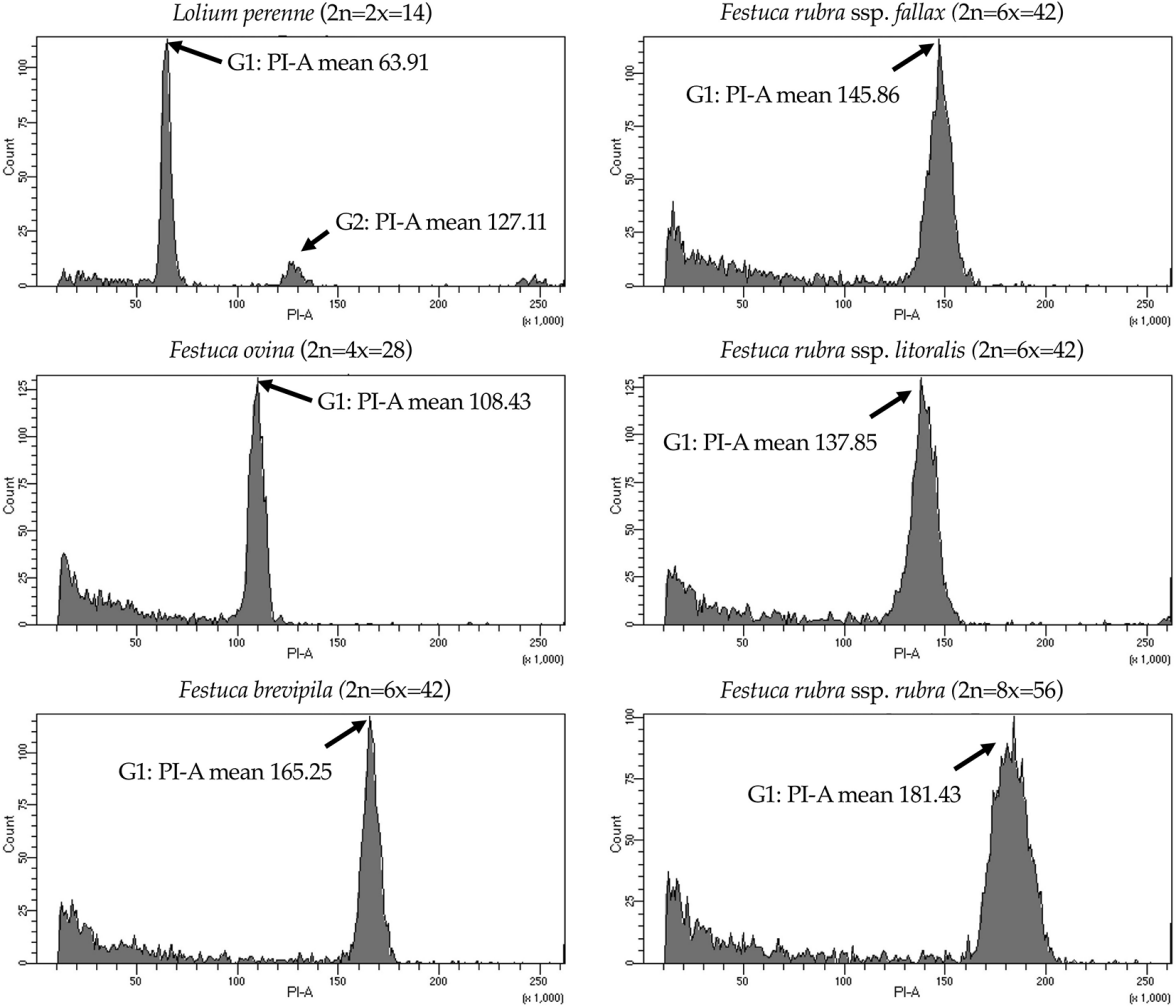
Flow cytometry results for the five fine fescue taxa. *Lolium perenne* (2n = 2x = 14) was used as the reference. Flow cytometry was able to separate *F. rubra* ssp. *rubra* from the other two taxa in the *F. rubra* complex. The mean PI-A values of *F. rubra* ssp. *fallax* and *F. rubra* ssp. *litoralis* were similar (145.86 to 137.85).

We used flow cytometry to estimate the ploidy levels of five fine fescue taxa by measuring the DNA content in each nucleus. DNA content was reflected by the flow cytometry mean PI-A value. Overall, fine fescue taxa had mean PI-A values roughly from 110 to 180 (**Figure 1** and **Figure S1**). *F. rubra* ssp. *rubra* cv. Navigator II (2n = 8x = 56) had the highest mean PI-A value (181.434, %rCV 4.4). *F. rubra* ssp. *litoralis* cv. Shoreline (2n = 6x = 42) and *F. rubra* ssp. *fallax* cv. Treazure II (2n = 6x = 42) had similar mean PI-A values of 137.852, %rCV 3.7 and 145.864, %rCV 3.5, respectively. *F. brevipila* cv. Beacon (2n = 6x = 42) had a mean PI-A of 165.25, %rCV 1.9, while *F. ovina* cv. Quatro (2n = 4x = 28) had a mean PI-A of 108.43, %rCV 2.9. Standard reference *L. perenne* cv. Artic Green (2n = 2x = 14) had a G1 phase mean PI-A of 63.91, %rCV 3.0. USDA *F. ovina* PI 230246 (2n = 2x = 14) had a G1 mean PI-A of 52.73 (histogram not shown). The estimated genome size of USDA PI 230246 was 4.67 pg/2C. Estimated ploidy level of *F. brevipila* cv. Beacon was 6.3, *F. ovina* cv. Quatro was 4.11, *F. rubra* ssp. *rubra* cv. Navigator II was 6.9, *F. rubra* ssp. *litoralis* cv. Shoreline was 5.2, and *F. rubra* ssp. *fallax* cv. Treazure II was 5.5 (**Table 1**). All newly estimated ploidy levels roughly correspond to previously reported ploidy levels based on chromosome counts.

**Table 1:** Summary of flow cytometry statistics, genome size, and ploidy level estimation of fine fescue species. *Lolium perenne* 2C DNA content was used to calculate fine fescue and USDA *F. ovina* PI 230246 genome size, and calculated PI 230246 DNA content was used as reference to infer fine fescue ploidy level.

Species name	Chromosome count	Cultivar name	Mean PI-A	%rCV*	Estimated genome size (pg/nuclei)	Estimated ploidy level
*F. brevipila*	2n = 6x = 42	Beacon	165.3	1.9	14.6	6.3
*F. ovina*	2n = 4x = 28	Quatro	108.4	2.9	9.6	4.1
*F. ovina* PI 230246	2n = 2x = 14	NA	52.7	3.1	4.7	1.7
*F. rubra* ssp. *rubra*	2n = 8x = 56	Navigator II	181.4	4.4	16.1	6.9
*F. rubra* ssp. *litoralis*	2n = 6x = 42	Shoreline	137.9	3.7	12.2	5.2
*F. rubra* ssp. *fallax*	2n = 6x = 42	Treazure II	145.9	3.5	12.9	5.5
*L. perenne*	2n = 2x = 14	Artic Green	63.9	3.0	5.7	2.0

*%rCV: Quality of laser alignment. Low %rCV suggests high resolution sensitivity.

### Plastid Genome Assembly and Annotation of Five Fescue Taxa

**Figure 2:**
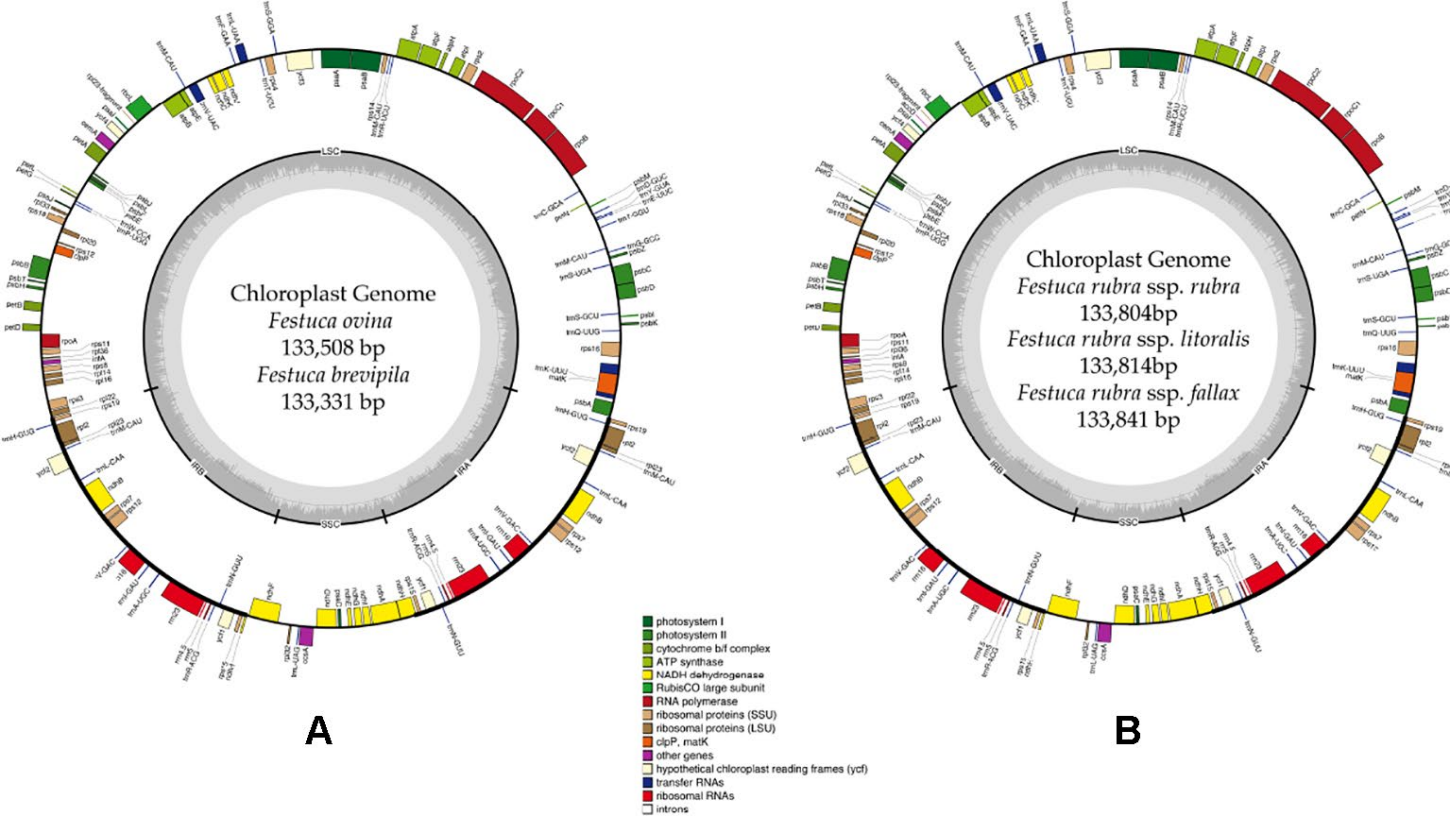
Whole chloroplast genome structure of *F. ovina* complex **(A)** and *F. rubra* complex **(B)**. Genes inside the circle are transcribed clockwise, and genes outside are transcribed counter-clockwise. Genes belong to different functional groups are color coded. GC content is represented by the dark gray inner circle, and the light gray corresponded to the AT content. IRA(B), inverted repeat A(B); LSC, large single copy region; SSC, small single copy region.

A total of 47,843,878 reads were produced from the five fine fescue taxa. After Illumina adaptor removal, we obtained 47,837,438 reads. The assembled chloroplast genomes ranged from 133,331 to 133,841 bp. The large single copy (LSC) and small single copy (SSC) regions were similar in size between the sequenced fine fescue accessions (78 kb and 12 kb, respectively). *F. ovina* and *F. brevipila* in the *F. ovina* complex had exactly the same size inverted repeat (IR) region (42,476 bp). In the *F. rubra* complex, *F. rubra* ssp. *rubra* and *F. rubra* ssp. *litoralis* had the same IR size (21,235 bp). Species in the *F. rubra* complex had a larger chloroplast genome size compared to species in the *F. ovina* complex. All chloroplast genomes shared similar GC content (38.4%) (**Figure 2**, **Table 2**). The fine fescue chloroplast genomes encoded for 113-114 genes, including 37 transfer RNAs (tRNA), 4 ribosomal RNAs (rRNA), and 72 protein-coding genes (**Table 2**). Genome structures were similar among all five fine fescue taxa sequenced, except that the pseudogene *accD* was annotated in all three subspecies of *F. rubra,* but not in the *F. ovina* complex (**Table S1**).

**Table 2:** Characteristics of fine fescue chloroplast genomes.

	*F. brevipila* cv. Beacon	*F. ovina* cv. Quatro	*F. rubra* ssp. *rubra* cv. Navigator II	*F. rubra* ssp. *litoralis* cv. Shoreline	*F. rubra* ssp. *fallax* cv. Treazure II
NCBI GenBank ID	MN309822	MN309824	MN309825	MN309823	MN309826
Total Genome Size (bp)	133,331	133,508	133,804	133,814	133,841
Large Single Copy (bp)	78,462	78,632	78,888	78,909	78,882
Small Single Copy (bp)	12,393	12,400	12,446	12,435	12,451
Inverted Repeat (bp)	42,476	42,476	42,470	42,470	42,508
Ratio of LSC (%)	58.85	58.9	58.96	58.97	58.94
Ratio of SSC (%)	9.29	9.29	9.3	9.29	9.3
Ratio of IRs (%)	31.86	31.82	31.74	31.74	31.76
GC content (%)	38.4	38.4	38.4	38.4	38.4

### Chloroplast Genome IR Expansion and Contraction

**Figure 3:**
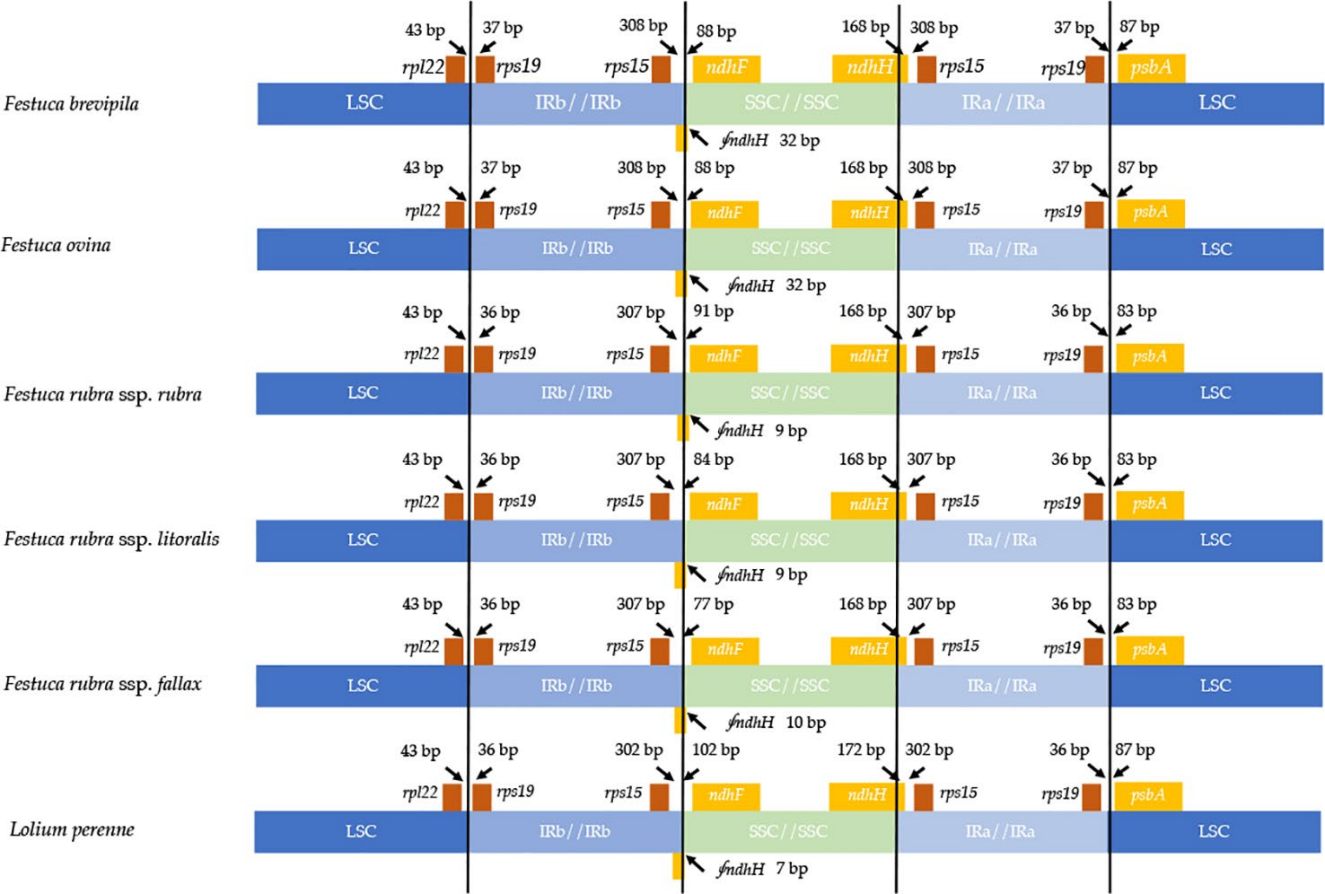
Comparison for border positions of LSC, SSC, and IR regions among five fine fescues and *L. perenne*. Genes are denoted by boxes, and the gap between the genes and the boundaries is indicated by the number of bases unless the gene coincides with the boundary. Extensions of genes are also indicated above the boxes.

Contraction and expansion of the IR regions resulted in the size variation of chloroplast genomes. We examined the four junctions in the chloroplast genomes, LSC/IRa, LSC/IRb, SSC/IRa, and SSC/IRb of the fine fescue and the model turfgrass species *L. perenne*. Although the chloroplast genome of fine fescue taxa was highly similar, some structural variations were still found in the IR/LSC and IR/SSC boundary. Similar to *L. perenne*, fine fescue taxa chloroplast genes *rpl22-rps19*, *rps19-psbA* were located in the junction of IR and LSC; *rps15-ndhF* and *ndhH-rps15* were located in the junction of IR/SSC. In the *F. ovina* complex, the *rps19* gene was 37 bp into the LSC/IRb boundary while in the *F. rubra* complex and *L. perenne*, the *rps19* gene was 36 bp into the LSC/IRb boundary (**Figure 3**). The *rsp15* gene was 308 bp from the IRb/SSC boundary in *F. ovina* complex, 307 bp in *F. rubra* complex, and 302 bp in *L. perenne*. Both the *ndhH* and the pseudogene fragment of the *ndhH* (⨚*ndhH)* genes spanned the junction of the IR/SSC. The ⨚*ndhH* gene crossed the IRb/SSC boundary with 32 bp into SSC in *F. brevipila* and *F. ovina*, 9 bp in *F. rubra* ssp. *rubra* and *F. rubra* ssp. *litoralis*, 10 bp in *F. rubra* ssp. *fallax*, and 7 bp in *L. perenne*. The *ndhF* gene was 88 bp from the IRb/SSC boundary in *F. brevipila* and *F. ovina*, 91 bp in *F. rubra* ssp. *rubra*, 84 bp in *F. rubra* ssp. *litoralis*, 77 bp in *F. rubra* ssp. *fallax*, and 102 bp in *L. perenne*. Finally, the *psbA* gene was 87 bp apart from the IRa/LSC boundary into the LSC in *L. perenne* and *F. ovina* complex taxa but 83 bp in the *F. rubra* complex taxa.

### Whole Chloroplast Genome Comparison and Repetitive Element Identification

**Figure 4:**
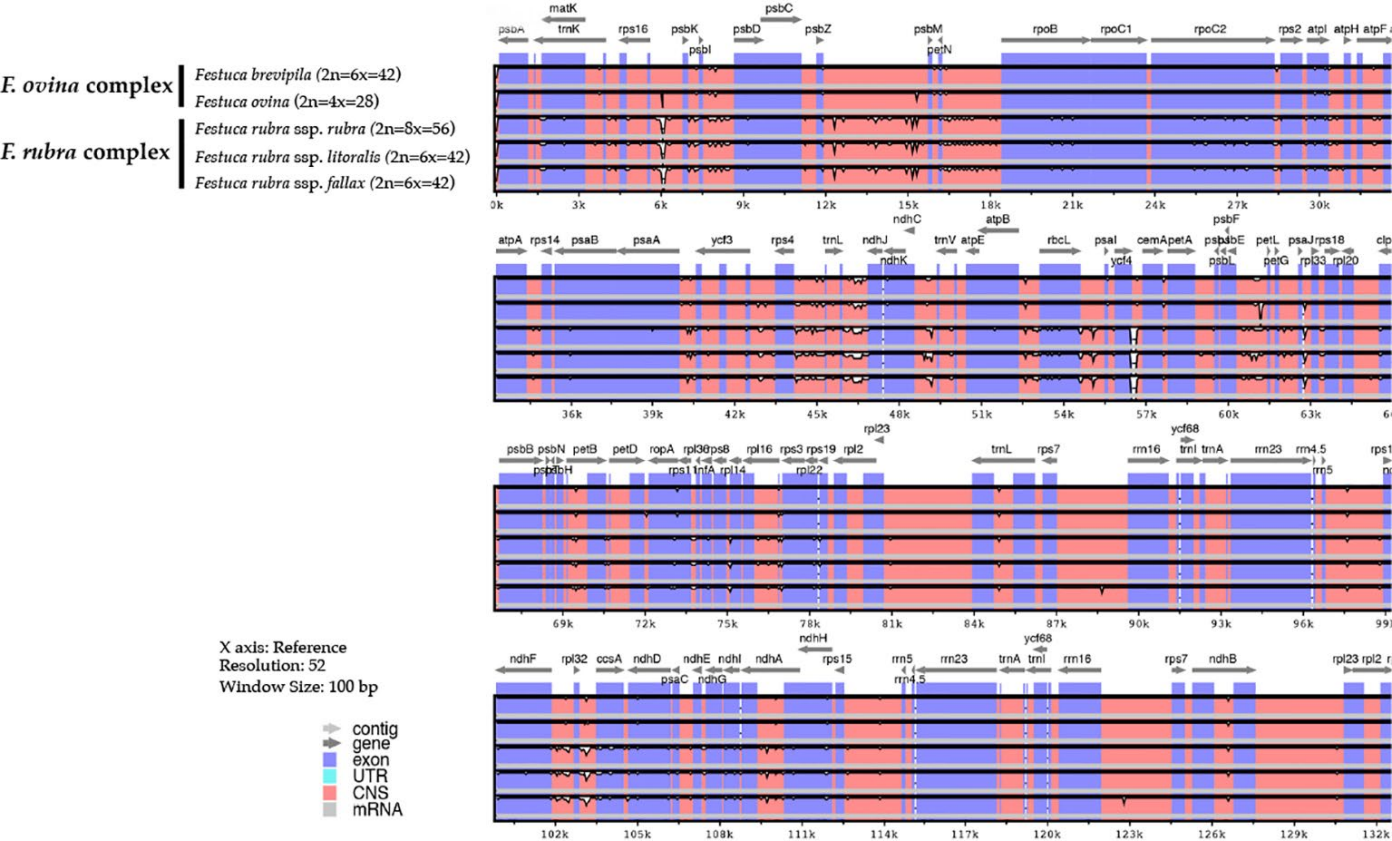
Sequence identity plot of fine fescues chloroplast genome sequences with *F. ovina* (2x) as the reference using mVISTA. A cut-off of 70% identify was used for the plots, and the percent of identity varies from 50% to 100% as noted on the y-axis. Most of the sequence variations between fine fescues were in intergenic regions. Taxa in the *F. ovina* complex, *F. brevipila*, and *F. ovina* showed high sequence similarity. Similarly, subspecies within *F. rubra* complex also showed high sequence similarity.

**Figure 5:**
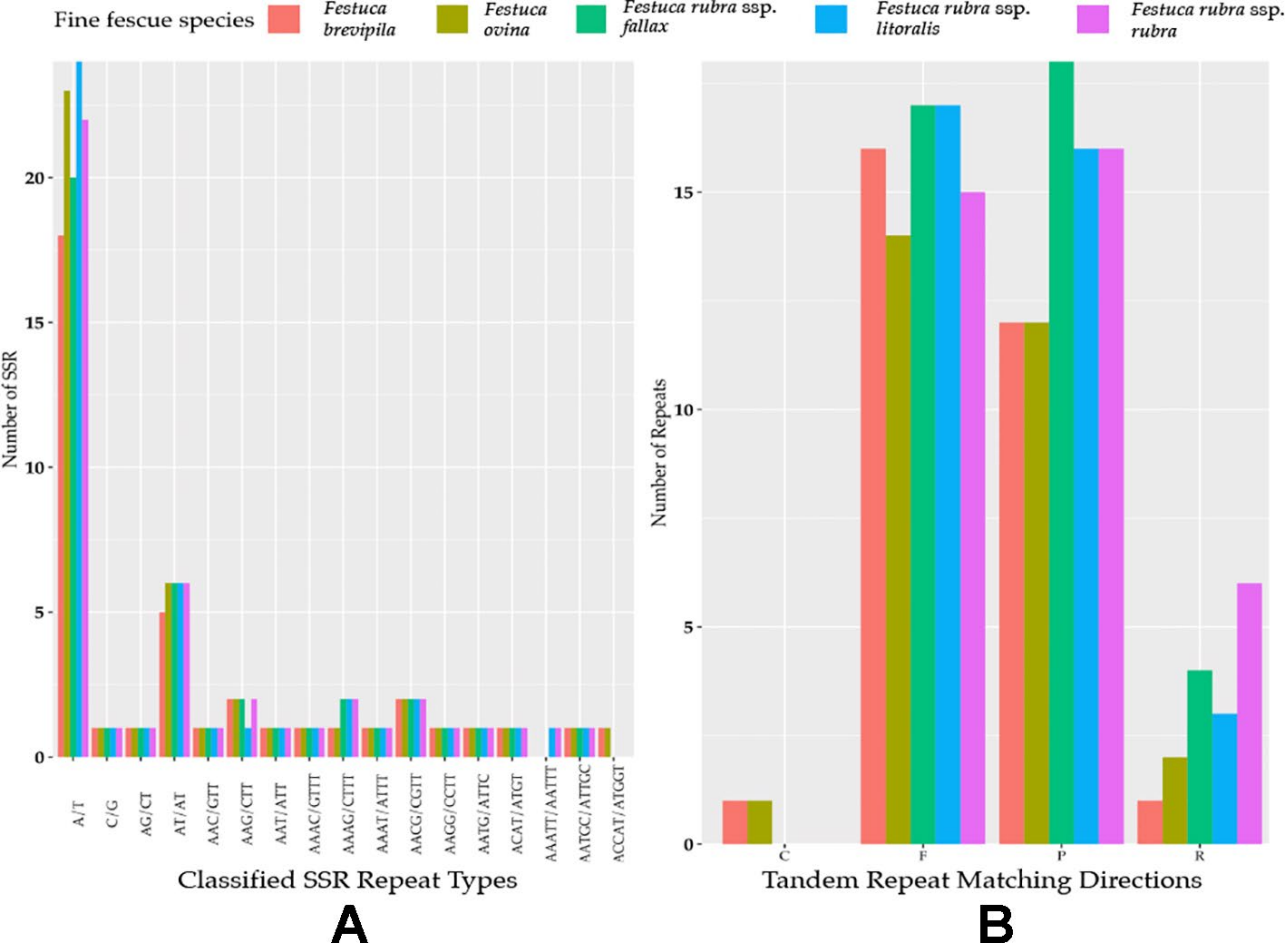
**(A)** SSR repeat type and numbers in the five fine fescue taxa sequenced. Single nucleotide repeat type has the highest frequency. No hexanucleotide repeats were identified in the fine fescue chloroplast genomes sequenced. One penta-nucleotide repeat type (AAATT/AATTT) is unique to *F. rubra* ssp. *rubra* and *F. rubra* ssp. *litoralis*. One penta-nucleotide repeat type (ACCAT/ATGGT) is unique to *F. brevipila* and *F. ovina*. **(B)** Long repeats sequences in fine fescue chloroplast genomes. Complement (C) match was only identified in the *F. ovina* complex. Reverse (R) match has the most number variation in fine fescues.

Genome-wide comparison among five fine fescue taxa showed high sequence similarity with most variations located in intergenic regions (**Figure 4**). To develop markers for species screening, we predicted a total of 217 SSR markers for fine fescue taxa sequenced (*F. brevipila* 39; *F. ovina* 45; *F. rubra* ssp. *rubra* 45; *F. rubra* ssp. *litoralis* 46; *F. rubra* ssp. *fallax* 42) that included 17 different repeat types for the fine fescue species (**Figure 5**, **Table S2**). The most frequent repeat type was A/T repeats, followed by AT/AT. The pentamer AAATT/AATTT repeat was only presented in the rhizomatous *F. rubra* ssp. *litoralis* and *F. rubra* ssp. *rubra*, while ACCAT/ATGGT was only found in *F. ovina* complex species *F. brevipila* and *F. ovina*. Similar to SSR loci prediction, we also predicted long repeats for the fine fescue species and identified a total of 171 repeated elements ranging in size from 20 to 51 bp (**Figure 5B**, **Table S3**). Complementary (C) matches were only identified in *F. brevipila* and *F. ovina*. *F. rubra* species had more palindromic (P) and reverse (R) matches. A number of forward (F) matches were similar between five taxa. Selected polymorphic regions were validated by PCR and gel electrophoresis assay (**Figure S2**).

### SNP and InDel Distribution in the Coding Sequence of Five Fine Fescue Species

To identify single nucleotide polymorphisms (SNPs, non-reference allele in this content), we used the diploid *F. ovina* chloroplast genome (JX871940.1) as the reference for the mapping and used the genome annotation file to identify genic and non-genic SNPs. The total genic and non-genic sequence of (JX871940.1) were 60,582 and 72,583 bp, respectively. We found SNP polymorphisms were over-present within intergenic regions in the *F. ovina* complex (∼0.3 SNP/Kbp more), while were under-present in the *F. rubra* complex (∼0.5 SNP/Kbp less). Most InDels were located in intergenic regions of the fine fescue species (**Table 3**). Between *F. ovina* and the *F. rubra* complex, *the ropC2* gene had the most SNPs (4 vs 31). The *rbcL* gene also has a high level of variation (1 vs 14.3). In addition, *rpoB*, *ccsA*, NADH dehydrogenase subunit genes (*ndhH*, *ndhF*, *ndhA*), and ATPase subunit genes (*atpA*, *atpB*, *aptF*) also showed variation between *F. ovina* and *F. rubra* complexes. Less SNP and InDel variation were found within each complex (**Table 3**, **Tables S4** and **S5**).

**Table 3:** Distribution of SNPs and InDels for the five fine fescue taxa sequenced in this study.

	*F. brevipila*	*F. ovina*	*F. rubra *ssp. *rubra*	*F. rubra* ssp. *litoralis*	*F. rubra* ssp. *fallax*
Total number of SNPs	98	134	638	615	624
SNPs in the coding region	35	52	306	301	300
SNPs in intergenic region	63	82	332	314	324
SNPs per Kbp in genic region	0.5777	0.8583	5.0510	4.9685	4.9520
SNPs per Kbp in non-genic region	0.8680	1.1297	4.5741	4.3261	4.4639
Total number of InDels	112	102	149	156	149
InDels in the coding region	22	17	27	26	27
InDels in intergenic region	90	85	122	130	122
Percentage of InDels in the intergenic region	80.36	83.33	81.88	83.33	81.88
Average sequencing depth	171.61	86.81	101.58	77.04	50.94

### Nucleotide Diversity Calculation

**Figure 6:**
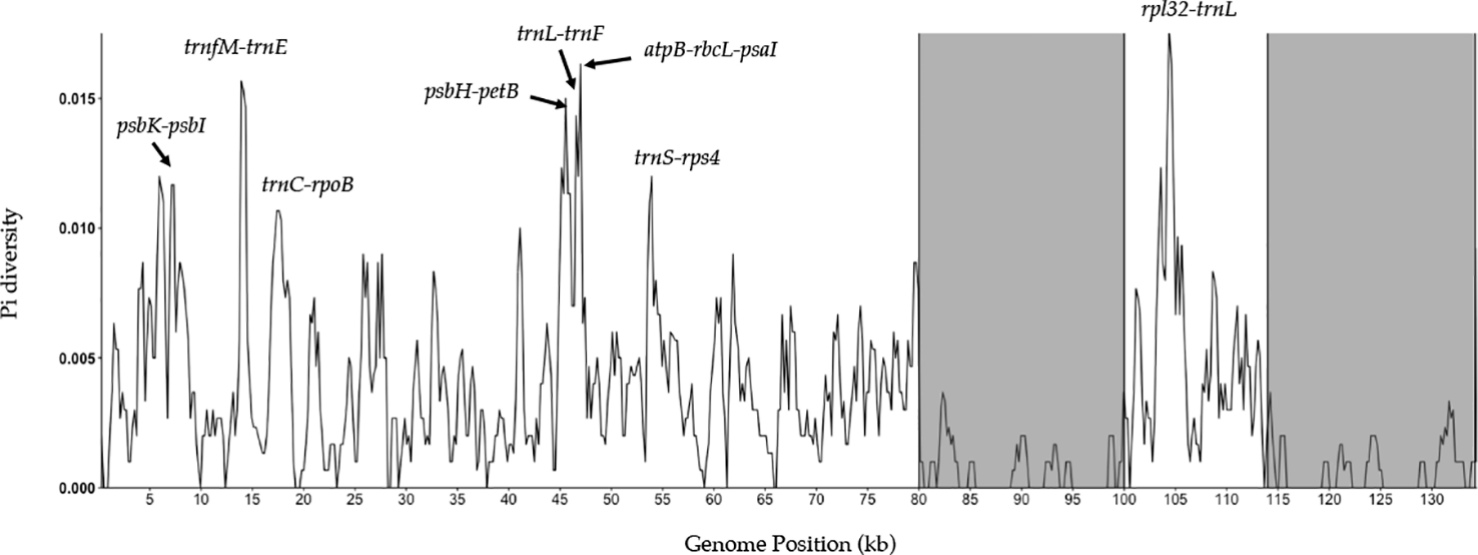
Sliding window analysis of fine fescue whole chloroplast genomes. Window size: 600 bp, step size: 200 bp. X-axis: the position of the midpoint of a window (kb). Y-axis: nucleotide diversity of each window. Inverted repeat regions are highlighted in gray. *rpl32-trnL* region has the most nucleotide diversity followed by *psbH- petB-trnL-trnF-trnS-rps4* region.

**Figure 7:**
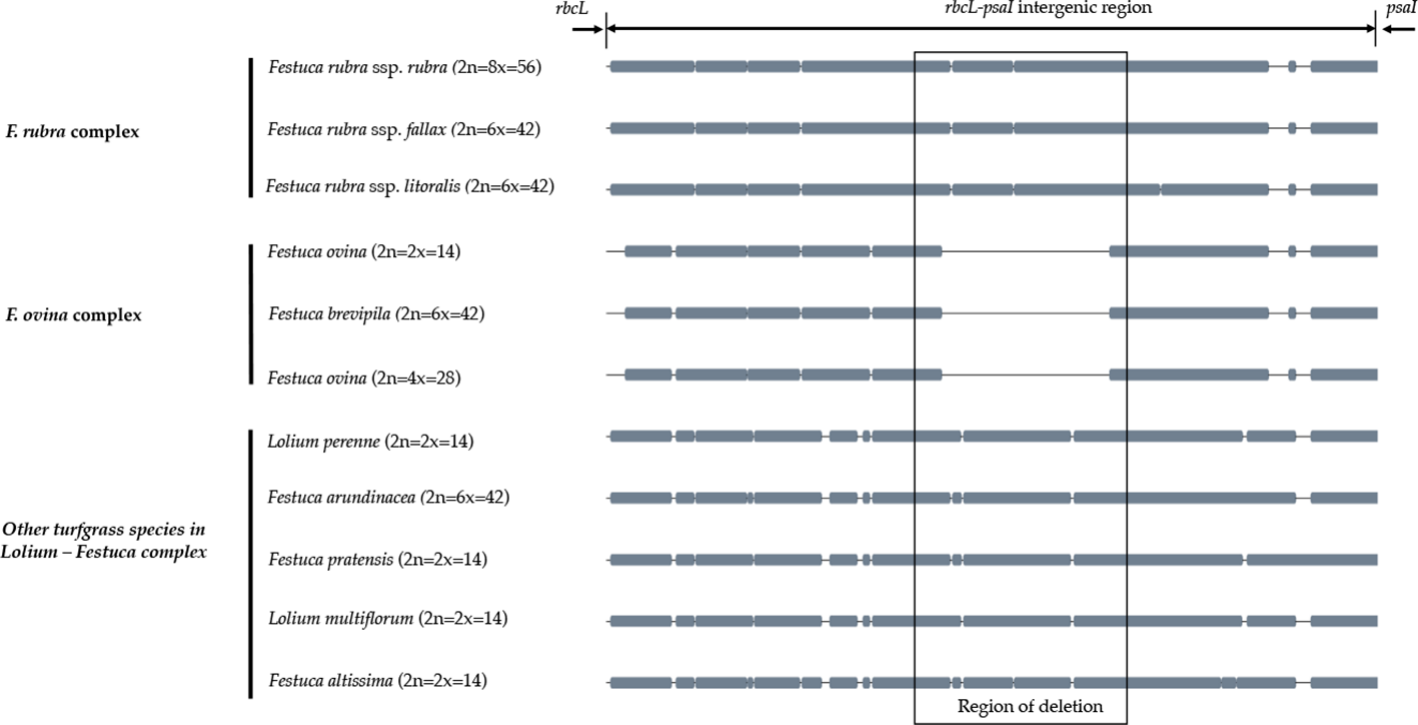
The alignment of *rbcL-psaI* intergenic sequence shows that the pseudogene *accD* is missing in both *F. ovina* (2x, 4x) and *F. brevipila* but present in the *F. rubra* complex and other species examined in this study. Species were ordered by complexes.

A sliding window analysis successfully detected highly variable regions in the fine fescue chloroplast genomes (**Figure 6**, **Table S6**). The average nucleotide diversity (Pi) among fine fescue taxa was relatively low (0.00318). The IR region showed lower variability than the LSC and SSC region. There were several divergent loci having a Pi value greater than 0.01 (*psbK-psbI*, *trnfM-trnE*, *trnC-rpoB*, *psbH-petB*, *trnL-trnF*, *trnS-rps4*, *aptB-rbcL-psaI*, and *rpl32-trnL*), but mostly within intergenic regions. The *rbcL-psaI* region contained a highly variable *accD-like* region in some fine fescue taxa, so we looked at the structural variation of 10 taxa in the *Festuca-Lolium* complex. We found taxa in broad-leaved fescue and *F. rubra* complex had similar structure, while *F. ovina* (2x, 4x) and *F. brevipila* had a 276 bp deletion in the *rbcL-psaI* intergenic region (**Figure 7**).

### Phylogenetic Reconstruction of Fine Fescue Species

**Figure 8:**
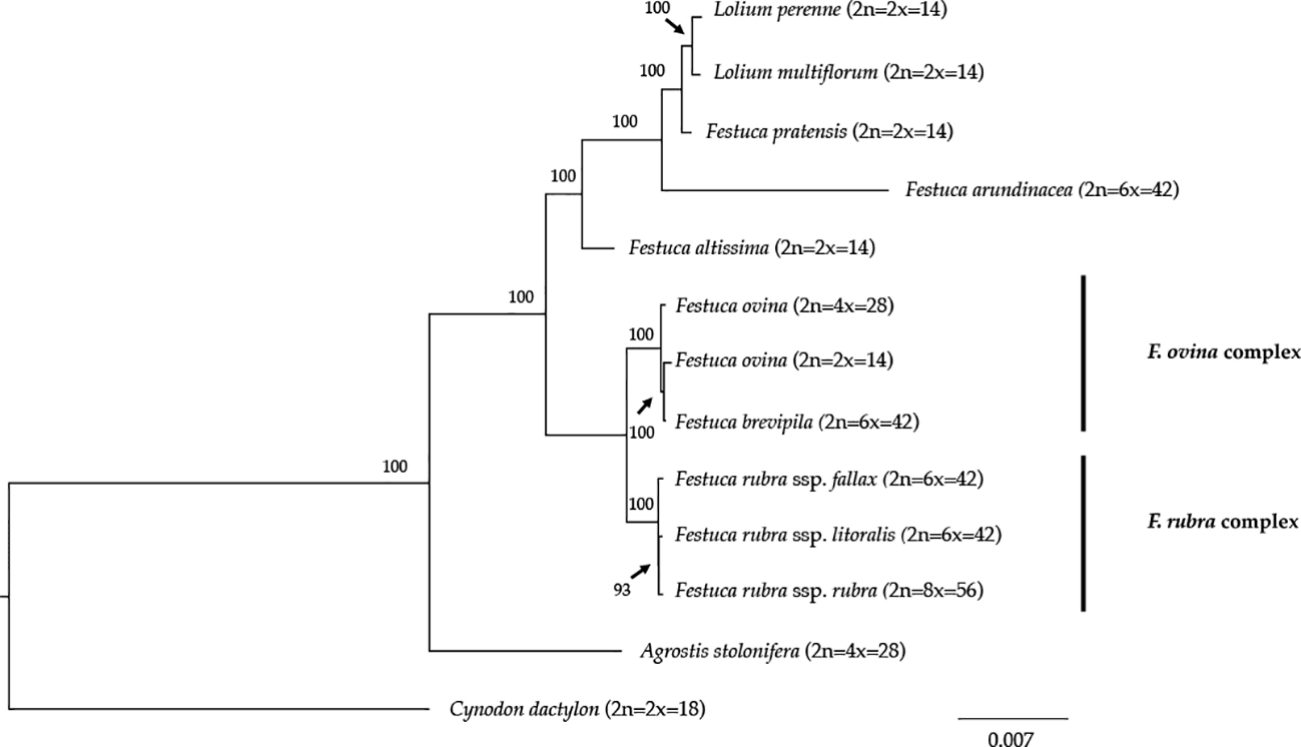
Maximum likelihood (ML) phylogram of the *Festuca-Lolium* complex with 1,000 bootstrap replicates. Fine fescues were grouped into previous named complexes (*F. ovina* and *F. rubra*), sister to broad leaved fescues in the *Festuca-Lolium* complex.

We reconstructed the phylogenetic relationships of taxa within the *Festuca-Lolium* complex using the chloroplast genomes sequenced in our study and eight publicly available complete chloroplast genomes including six taxa within the *Festuca-Lolium* complex (**Figure 8**). The dataset included 125,824 aligned characters, of which 3,923 were parsimony-informative and 91.11% characters are constant. The five fine fescue taxa were split into two clades ([ML]BS = 100). In the *F. ovina* complex, two *F. ovina* accessions included in the phylogenetic analysis, a diploid one from GenBank, and a tetraploid one newly sequenced in this study are paraphyletic to *F. brevipila* ([ML]BS = 100). All three subspecies of *F. rubra* formed a strongly supported clade ([ML]BS = 100). Together they are sisters to the *F. ovina* complex ([ML]BS = 100).

## Discussion

In this study, we used flow cytometry to determine the ploidy level of five fine fescue cultivars, assembled the chloroplast genomes for each, and identified structural variation and mutation hotspots. We also identified candidate loci for marker development to facilitate fine fescue species identification. Additionally, we reconstructed the phylogenetic relationships of the *Festuca-Lolium* complex using plastid genome information generated in this study along with other publicly-available plastid genomes.

While most crop plants are highly distinctive from their close relatives, *Festuca* is a species-rich genus that contains species with highly similar morphology and different ploidy level. Consequently, it is difficult for researchers to interpret species identity. In our case, flow cytometry was able to successfully separate fine fescue taxa *F. brevipila* cv. Beacon, *F. ovina* cv. Quatro and *F. rubra* ssp. *rubra* cv. Navigator II based on the estimated ploidy levels. However, it is difficult to distinguish between *F. rubra* ssp. *litoralis* cv. Shoreline and *F. rubra* ssp. *fallax* cv. Treazure II as they had similar PI-A values based on flow cytometry.

We noticed that the average mean PI-A of the diploid *L. perenne* (63.91) was higher than the mean PI-A of diploid *F. ovina* (52.73), suggesting that *F. ovina* taxa have smaller genome size than *L. perenne*. The ploidy estimation in the *F. ovina* complex is fairly consistent, while the estimations of genome sizes in the *F. rubra* complex are smaller than we expected, even though these two complexes are closely related. Indeed, a similar finding was reported by Huff et al. (Huff and Palazzo, 1998) who reported that *F. brevipila* has a larger genome size than *F. rubra* ssp. *litoralis* and *F. rubra* ssp. *fallax*, both of which have the same ploidy level as *F. brevipila*. The range of variation in DNA content within each complex suggest a complicated evolutionary history in addition to polyploidization (Huff and Palazzo, 1998).

When we cannot identify taxon based on the ploidy level, we need different approaches to identify them. The presence and absence of rhizome formation could be taken into consideration; for example, *F. rubra* ssp. *fallax* cv. Treazure II is a bunch type turfgrass, while *F. rubra* ssp. *litoralis* cv. Shoreline forms short and slender rhizomes (Meyer and Funk, 1989). This method may not be effective because rhizome formation can be greatly affected by environmental conditions (Yang et al., 2015; Ma and Huang, 2016).

To further develop molecular tools to facilitate species identification, we carried out chloroplast genome sequencing. We assembled the complete chloroplast genomes of five low-input turfgrass fine fescues using Illumina sequencing. Overall, the chloroplast genomes had high sequence and structure similarity among all five fine fescue taxa sequenced, especially within each complex. All five chloroplast genomes share similar gene content except for the three species in the *F. rubra* complex that have a pseudogene Acetyl-coenzyme A carboxylase carboxyl transferase subunit (*accD*). The *accD* pseudogene is either partially or completely absent in some monocots. Instead, a nuclear-encoded ACC enzyme has been found to replace the plastic *accD* gene function in some angiosperm linage (Rousseau-Gueutin et al., 2013). Indeed, even though the *accD* pseudogene is missing in the *F. brevipila* chloroplast genome, the gene transcript was identified in a transcriptome sequencing dataset (unpublished data), suggesting that this gene has been translocated to nucleus genome. Previous studies have shown that broad-leaf fescues, *L. perenne*, *O. sativa*, and *H. vulgare* all had the pseudogene *accD* gene, while it was absent in diploid *F. ovina*, *Z. mays*, *S. bicolor*, *T. aestivum*, and *B. distachyon* (Hand et al., 2013). Broad-leaf and fine-leaf fescues diverged around 9 Mya ago (Fjellheim et al., 2006), which raises an interesting question about the mechanisms of the relocation of *accD* among closely related taxa in the *Festuca-Lolium* complex and even within fine fescue species.

In plants, chloroplast genomes are generally considered "single copy" and lack recombination due to maternal inheritance (Ebert and Peakall, 2009). For this reason, chloroplast genomes are convenient for developing genetic markers for distinguishing species and subspecies. We have identified a number of repeat signatures that are unique to a single species or species complex in fine fescue. For example, complement match is only identified in *F. ovina* complex, and *F. rubra* complex has more reversed matches. We also identified two SSR repeats unique to each of the two complexes. The first one consists of AAATT/AATTT repeat units is unique to *F. rubra* ssp. *litoralis* and *F. rubra* ssp. *rubra*, and the second one consists of ACCAT/ATGGT repeat units is unique to *F. brevipila* and *F. ovina*. In cases like the identification of hexaploids *F. brevipila*, *F. rubra* ssp. *fallax*, and *F. rubra* ssp*. litoralis*, it is critical to have these diagnostic repeats given all three taxa share similar PI-A values from flow cytometry. Taxon-specific tandem repeats could also aid the SSR repeats for species identification. We used chloroplast sequence developed candidate primer sets to solve the problem. Primer set (1) provided a clear separation of *F. rubra* ssp. *litoralis* cv. Shoreline and *F. rubra* ssp. *fallax* cv. Treazure II when flow cytometry was not able to separate them. Primer set (2) provided clear separation of *F. brevipila* cv. Beacon and *F. ovina* cv. Quatro, which provided an alternative method for *F. ovina* complex taxa identification. By combining both flow cytometry and candidate primer sets developed in this study, researchers will be able to identify fine fescue taxa within and between two complexes.

Nucleotide diversity analysis suggested that several variable genome regions exist among the five fine fescue taxa sequenced in this study. These variable regions included previously known highly variable chloroplast regions such as *trnL-trnF* and *rpl32-trnL* (Demesure et al., 1995; Dong et al., 2012). These regions have undergone rapid nucleotide substitution and are potentially informative molecular markers for characterization of fine fescue species.

Phylogeny inferred from DNA sequence is valuable for understanding the evolutionary context of a species. The phylogenetic relationship of fine fescue using whole plastid genome sequences agrees with previous classification based on genome size estimation and morphology (Huff and Palazzo, 1998; Cheng et al., 2016). The *F. ovina* complex includes *F. ovina* and *F. brevipila* and the *F. rubra* complex includes *F. rubra* ssp. *rubra*, *F. rubra* ssp*. litoralis* and *F. rubra* ssp. *fallax*, with the two rhizomatous subspecies (ssp. *rubra* and ssp. *literalis*) being sister to each other. Within the *Festuca-Lolium* complex, fine fescues are monophyletic and together sister to a clade consists of broad-leaved fescues and *Lolium*. In our analysis, *F. brevipila* (6x) is nested within *F. ovina* and sister to the diploid *F. ovina*. It is likely that *F. brevipila* arose from the hybridization between *F. ovina* (2x) and *F. ovina* (4x). Considering the complex evolutionary history of this genus, further research using nuclear loci sequences are needed to provide a more accurate phylogeny tree and validate this hypothesis.

The diversity of fine fescue provides valuable genetic diversity for breeding and cultivar development. Breeding fine fescue cultivars for better disease resistance, heat tolerance, and traffic tolerance could be achieved through screening wild accessions and by introgressing desired alleles into elite cultivars. Some work has been done using *Festuca* accessions in the USDA Germplasm Resources Information Network (GRIN) (https://www.ars-grin.gov) to breed for improved forage production in fescue species (Robbins et al., 2016). To date, there are 229 *F. ovina* and 486 *F. rubra* accessions in the USDA GRIN. To integrate this germplasm into breeding programs, plant breeders and other researchers need to confirm the ploidy level using flow cytometry and further identify them using molecular markers. Resources developed in this study could provide the tools to screen the germplasm accessions and refine the species identification so breeders can efficiently use these materials for breeding and genetics improvement of fine fescue species.

## Materials and Methods

### Plant Material

Seeds from the fine fescue cultivars were obtained from the 2014 National Turfgrass Evaluation Program (www.ntep.org, USA) and planted in the Plant Growth Facility at the University of Minnesota, St. Paul campus under 16 h daylight (25°C) and 8 h dark (16°C) with weekly fertilization. Single genotypes of *F. brevipila* cv. Beacon, *F. rubra* ssp. *litoralis* cv. Shoreline, *F. rubra* ssp. *rubra* cv. Navigator II, *F. rubra* ssp. *fallax* cv. Treazure II, and *F. ovina* cv. Quatro were selected and used for chloroplast genome sequencing.

### Flow Cytometry

To determine the ploidy level of the cultivars used for sequencing and compare them to previous work (2n = 4x = 28: *F. ovina*; 2n = 6x = 42: *F. rubra* ssp. *litoralis*, *F. rubra* ssp. *fallax*, and *F. brevipila*; 2n = 8x = 56: *F. rubra* ssp. *rubra*), flow cytometry was carried out using *Lolium perenne* cv. Artic Green (2n = 2x = 14) as the reference. Samples were prepared using CyStain PI Absolute P (Sysmex, product number 05-5022). Briefly, to prepare the staining solution for each sample, 12 µl propidium iodide (PI) was added to 12 ml of Cystain UV Precise P staining buffer with 6 µl RNase A. To prepare plant tissue, a total size of 0.5 cm x 0.5 cm leaf sample of the selected fine fescue was excised into small pieces using a razor blade in 500 µl CyStain UV Precise P extraction buffer and passed through a 50 µm size filter (Sysmex, product number 04-004-2327). The staining solution was added to the flow-through to stain nuclei in each sample. Samples were stored on ice before loading the flow cytometer. Flow cytometry was carried out using the BD LSRII H4760 (LSRII) instrument with PI laser detector using 480V with 2,000 events at the University of Minnesota Flow Cytometry Resource (UCRF). Data was visualized and analyzed on BD FACSDiva 8.0.1 software. To estimate the genome size, *L. perenne* DNA (5.66 pg/2C) was used as standard (Arumuganathan et al., 1999), and USDA PI 230246 (2n = 2x = 14) was used as diploid fine fescue relative (unpublished data). To infer fine fescues ploidy, estimation was done using equations (1) and (2) (Doležel et al., 2007).

(1) $$\text{Sample 2C DNA Content}=\text{Standard 2C DNA Content}\left(\text{pg DNA}\right)\times \frac{\left(\text{Sample G1 Peak Mean}\right)}{\left(\text{Standard G1 Peak Mean}\right)}$$

(2) $$\text{Sample Ploidy}=\frac{2\text{n}\times \text{Sample pg/Nucleus}}{\text{Diploid Relative pg/Nucleus}}$$

### DNA Extraction and Sequencing

To extract DNA for chloroplast genome sequencing, 1 g of fresh leaves was collected from each genotype and DNA was extracted using the Wizard Genomic DNA Purification Kit (Promega, USA) following manufacturer instructions. DNA quality was examined on 0.8% agarose gel and quantified *via* PicoGreen (Thermo Fisher, Catalog number: P11496). Sequencing libraries were constructed by NovoGene, Inc. (Davis, CA) using Nextera XT DNA Library Preparation Kit (Illumina) and sequenced in 150 bp paired-end mode, using the HiSeq X Ten platform (Illumina Inc., San Diego, CA, USA) with an average of 10 million reads per sample. All reads used in this study were deposited in the NCBI Sequence Read Archive (SRA) under BioProject PRJNA512126.

### Chloroplast Genome Assembly and Annotation

Raw reads were trimmed of Illumina adaptor sequences using Trimmomatic (v. 0.32) (Bolger et al., 2014). Chloroplast genomes were *de novo* assembled using NovoPlasty v. 2.0 (Dierckxsens et al., 2016). Briefly, *rbcL* gene sequence from diploid *F. ovina* (NCBI accession number: JX871940) was extracted and used as the seed to initiate the assembly. NovoPlasty assembler configuration was set as follows: *k-mer* size = 39; insert size = auto; insert range = 1.8; and insert range strict 1.3. Reads with quality score above 25 were used to complete the guided assembly using *F. ovina* (NCBI accession number: JX871940) as the reference. Assembled plastid genomes for each taxon were manually corrected by inspecting the alignments of reads used in the assembly. The assembled chloroplast genomes were deposited under BioProject PRJNA512126, GenBank accession numbers MN309822-MN309826.

The assembled chloroplast genomes were annotated using the GeSeq pipeline (Tillich et al., 2017) and corrected using DOGMA online interface (https://dogma.ccbb.utexas.edu) (Wyman et al., 2004). BLAT [a BLAST-like alignment tool (Kent, 2002)] protein, tRNA, rRNA, and DNA search identity threshold was set at 80% in the GeSeq pipeline using the default reference database with the generate codon-based alignments option turned on. tRNAs were also predicted *via* tRNAscan-SE v2.0 and ARAGORN v 1.2.38 using the bacterial/plant chloroplast genetic code (Lowe and Eddy, 1997; Laslett and Canback, 2004). The final annotation was manually inspected and corrected using results from both pipelines. The circular chloroplast map was drawn by the OrganellarGenomeDRAW tool (OGDRAW) (Lohse et al., 2007).

### Nucleotide Polymorphism of Fine Fescue Species

To identify genes with the most single nucleotide polymorphism, quality trimmed sequencing reads of the five fine fescues were mapped to the diploid *F. ovina* chloroplast genome (NCBI accession number: JX871940) using BWA v.0.7.17 (Li and Durbin, 2009). SNPs and short indels were identified using bcftools v.1.9 with the setting "mpileup -Ou" and called *via* bcftools using the -mv function (Quinlan and Hall, 2010). Raw SNPs were filtered using bcftools filter -s option to filter out SNPs with low quality (Phred score cutoff 20, coverage cutoff 20). The subsequent number of SNPs per gene and InDel number per gene was calculated using a custom perl script SNP_vcf_from_gene_gff.pl (https://github.com/qiuxx221/fine-fescue-). 

To identify SSR markers for plant identification, MIcroSAtellite identification tool (MISA v 1.0) was used with a threshold of 10, 5, 4, 3, 3, and 3 repeat units for mono-, di-, tri-, tetra-, penta-, and hexanucleotide SSRs, respectively (Thiel et al., 2003). The identification of repetitive sequences and structure of whole chloroplast genome was done *via* REPuter program online server (https://bibiserv.cebitec.uni-bielefeld.de/reputer) (Kurtz et al., 2001). Program configuration was set with minimal repeat size set as 20 bp and with sequence identify above 90%. Data was visualized using ggplot2 in R (v 3.5.3). Finally, the sliding window analysis was performed using DnaSP (v 5) with a window size of 600 bp, step size 200 bp to detected highly variable regions in the fine fescue chloroplast genome (Librado and Rozas, 2009).

### Comparative Chloroplast Genomics Analysis

To compare fine fescue species chloroplast genome sequence variations, the five complete chloroplast genomes were aligned and visualized using mVISTA, an online suite of computation tools with LAGAN mode (Brudno et al., 2003; Frazer et al., 2004). The diploid *F. ovina* (NCBI accession number: JX871940) chloroplast genome and annotation were used as the template for the alignment.

### Phylogenetic Analysis of Fine Fescues and Related Festuca Species

To construct the phylogenetic tree of the fine fescues using the whole chloroplast genome sequence, chloroplast genomes of eight species were downloaded from GenBank. Of the eight downloaded genomes, perennial ryegrass (*Lolium perenne*, AM777385), Italian ryegrass (*Lolium multiflorum*, JX871942), diploid *F. ovina* (JX871940), tall fescue (*Festuca arundiancea*, FJ466687), meadow fescue (*F. pratensis*, JX871941), and wood fescue (*Festuca altissima*, JX871939) were within the *Festuca-Lolium* complex. Turfgrass species outside of *Festuca-Lolium* complex including creeping bentgrass (*Agrostis stolonifera* L., EF115543) and *Cynodon dactylon* (KY024482.1) were used as an outgroup. All chloroplast genomes were aligned using the MAFFT program (v 7) (Katoh and Standley, 2013); alignments were inspected and manually adjusted. Maximum likelihood (ML) analyses was performed using the RAxML program (v 8.2.12) under GTR+G model with 1,000 bootstrap (Stamatakis, 2006). The phylogenetic tree was visualized using FigTree (v 1.4.3) (https://github.com/rambaut/figtree) (Rambaut, 2012).

## Conclusions

Five newly-sequenced complete chloroplast genomes of fine fescue taxa were reported in this study. Chloroplast genome structure and gene contents are both conserved, with the presence and absence of *accD* pseudogene being the biggest structural variation between the *F. ovina* and the *F. rubra* complexes. We identified SSR repeats and long sequence repeats of fine fescues and discovered several unique repeats for marker development. The phylogenetic constructions of fine fescue species in the *Festuca-Lolium* complex suggested a robust and consistent relationship compared to the previous identification using flow cytometry. This information provided a reference for future fine fescue taxa identification.

## Data Availability Statement

The datasets generated for this study can be found in the NCBI Bioproject PRJNA512126.

## Author Contributions

YQ performed the experiments, analyzed the data, and wrote the manuscript. CH helped analyze data, wrote perl scripts. YY helped with phylogenetic analysis. EW secured funding for this project, supervised this research, provided suggestions, and comments. All authors contributed to the revision of the manuscript and approved the final version.

## Funding

This research is funded by the National Institute of Food and Agriculture, U.S. Department of Agriculture, Specialty Crop Research Initiative under award number 2017-51181-27222.

## Conflict of Interest

The authors declare that the research was conducted in the absence of any commercial or financial relationships that could be construed as a potential conflict of interest.

## References

[B1] Arumuganathan K. Tallury S. Fraser M. Bruneau A. Qu R. (1999). Nuclear DNA content of thirteen turfgrass species by flow cytometry. Crop Sci. 39, 1518–1521. 10.2135/cropsci1999.3951518x

[B2] Baldwin B. G. Sanderson M. J. Porter J. M. Wojciechowski M. F. Campbell C. S. Donoghue M. J. (1995). The ITS region of nuclear ribosomal DNA: a valuable source of evidence on angiosperm phylogeny. Ann. Mo. Bot. Garden, 82 (2), 247–277. 10.2307/2399880

[B3] Beard J. B. (1972). Turfgrass: Science and culture.

[B4] Bolger A. M. Lohse M. Usadel B. (2014). Trimmomatic: a flexible trimmer for Illumina sequence data. Bioinform. 30, 2114–2120. 10.1093/bioinformatics/btu170 PMC4103590 24695404

[B5] Bonos S. A. Huff D. R. (2013). Cool-season grasses: Biology and breeding. Turfgrass. Biology, Use Manage 7, 591–660. 10.2134/agronmonogr56.c17

[B6] Brudno M. Do C. B. Cooper G. M. Kim M. F. Davydov E. Green E. D. (2003). LAGAN and Multi-LAGAN: efficient tools for large-scale multiple alignment of genomic DNA. Genome Res. 13, 721–731. 10.1101/gr.926603 12654723 PMC430158

[B7] Bryan G. Mcnicoll J. Ramsay G. Meyer R. De Jong W. (1999). Polymorphic simple sequence repeat markers in chloroplast genomes of Solanaceous plants. Theor. Appl. Genet. 99, 859–867. 10.1007/s001220051306

[B8] Cahoon A. B. Sharpe R. M. Mysayphonh C. Thompson E. J. Ward A. D. Lin A. (2010). The complete chloroplast genome of tall fescue (Lolium arundinaceum; Poaceae) and comparison of whole plastomes from the family Poaceae. Am. J. Bot. 97, 49–58. 10.3732/ajb.0900008 21622366

[B9] Casler M. D. (2003). Turfgrass biology, genetics, and breeding. John Wiley & Sons.

[B10] Cheng T. Xu C. Lei L. Li C. Zhang Y. Zhou S. (2016). Barcoding the kingdom Plantae: new PCR primers for ITS regions of plants with improved universality and specificity. Mol. Ecol. Resour. 16, 138–149. 10.1111/1755-0998.12438 26084789

[B11] Clayton W. D. Renvoize S. A. (1986). Genera graminum. Grasses of the world. Genera graminum. Grasses of the World. 13.

[B12] Demesure B. Sodzi N. Petit R. (1995). A set of universal primers for amplification of polymorphic non-coding regions of mitochondrial and chloroplast DNA in plants. Mol. Ecol. 4, 129–134. 10.1111/j.1365-294X.1995.tb00201.x 7711952

[B13] Diekmann K. Hodkinson T. R. Wolfe K. H. Van Den Bekerom R. Dix P. J. Barth S. (2009). Complete chloroplast genome sequence of a major allogamous forage species, perennial ryegrass (Lolium perenne L.). DNA Res. 16, 165–176. 10.1093/dnares/dsp008 19414502 PMC2695775

[B14] Dierckxsens N. Mardulyn P. Smits G. (2016). NOVOPlasty: *de novo* assembly of organelle genomes from whole genome data. Nucleic Acids Res. 45, e18–e18. 10.1093/nar/gkw955 PMC5389512 28204566

[B15] Doležel J. Greilhuber J. Suda J. (2007). Estimation of nuclear DNA content in plants using flow cytometry. Nat. Protoc. 2, 2233. 10.1038/nprot.2007.310 17853881

[B16] Dong W. Liu J. Yu J. Wang L. Zhou S. (2012). Highly variable chloroplast markers for evaluating plant phylogeny at low taxonomic levels and for DNA barcoding. PloS One 7, e35071. 10.1371/journal.pone.0035071 22511980 PMC3325284

[B17] Ebert D. Peakall R. (2009). Chloroplast simple sequence repeats (cpSSRs): technical resources and recommendations for expanding cpSSR discovery and applications to a wide array of plant species. Mol. Ecol. Resour. 9, 673–690. 10.1111/j.1755-0998.2008.02319.x 21564725

[B18] Fjellheim S. Rognli O. A. Fosnes K. Brochmann C. (2006). Phylogeographical history of the widespread meadow fescue (Festuca pratensis Huds.) inferred from chloroplast DNA sequences. J. Biogeography 33, 1470–1478. 10.1111/j.1365-2699.2006.01521.x

[B19] Frazer K. A. Pachter L. Poliakov A. Rubin E. M. Dubchak I. (2004). VISTA: computational tools for comparative genomics. Nucleic Acids Res. 32, W273–W279. 10.1093/nar/gkh458 15215394 PMC441596

[B20] Hand M. L. Spangenberg G. C. Forster J. W. Cogan N. O. (2013). Plastome sequence determination and comparative analysis for members of the Lolium-Festuca grass species complex. G3. Genes Genomes Genet. 3 (4), 607–616. 10.1534/g3.112.005264 PMC3618348 23550121

[B21] Hebert P. D. Cywinska A. Ball S. L. Dewaard J. R. (2003). Biological identifications through DNA barcodes. Proc. R. Soc. London Ser. B.: Biol. Sci. 270, 313–321. 10.1098/rspb.2002.2218 PMC1691236 12614582

[B22] Huang Y.-Y. Cho S.-T. Haryono M. Kuo C.-H. (2017). Complete chloroplast genome sequence of common bermudagrass (Cynodon dactylon (L.) Pers.) and comparative analysis within the family Poaceae. PloS One 12, e0179055. 10.1371/journal.pone.0179055 28617867 PMC5472289

[B23] Hubbard C. E. (1968). Grasses. A guide to their structure, identification, uses, and distribution in the British Isles. Grasses. A guide to their structure, identification, uses, and distribution in the British Isles.

[B24] Huff D. R. Palazzo A. J. (1998). Fine fescue species determination by laser flow cytometry. Crop Sci. 38, 445–450. 10.2135/cropsci1998.0011183X003800020029x

[B25] Jenkin T. J. (1959). Fescue Species (Festuca L.). In: Roemer T. Rudorf W. Handbuch der Pflanzenzüchtung, 418-434.

[B26] Katoh K. Standley D. M. (2013). MAFFT multiple sequence alignment software version 7: improvements in performance and usability. Mol. Biol. Evol. 30, 772–780. 10.1093/molbev/mst010 23329690 PMC3603318

[B27] Kent W. J. (2002). BLAT-the BLAST-like alignment tool. Genome Res. 12, 656–664. 10.1101/gr.229202 11932250 PMC187518

[B28] Kurtz S. Choudhuri J. V. Ohlebusch E. Schleiermacher C. Stoye J. Giegerich R. (2001). REPuter: the manifold applications of repeat analysis on a genomic scale. Nucleic Acids Res. 29, 4633–4642. 10.1093/nar/29.22.4633 11713313 PMC92531

[B29] Laslett D. Canback B. (2004). ARAGORN, a program to detect tRNA genes and tmRNA genes in nucleotide sequences. Nucleic Acids Res. 32, 11–16. 10.1093/nar/gkh152 14704338 PMC373265

[B30] Li H. Durbin R. (2009). Fast and accurate short read alignment with Burrows-Wheeler transform. Bioinform. 25, 1754–1760. 10.1093/bioinformatics/btp324 PMC2705234 19451168

[B31] Librado P. Rozas J. (2009). DnaSP v5: a software for comprehensive analysis of DNA polymorphism data. Bioinform. 25, 1451–1452. 10.1093/bioinformatics/btp187 19346325

[B32] Lohse M. Drechsel O. Bock R. (2007). OrganellarGenomeDRAW (OGDRAW): a tool for the easy generation of high-quality custom graphical maps of plastid and mitochondrial genomes. Curr. Genet. 52, 267–274. 10.1007/s00294-007-0161-y 17957369

[B33] Lowe T. M. Eddy S. R. (1997). tRNAscan-SE: a program for improved detection of transfer RNA genes in genomic sequence. Nucleic Acids Res. 25, 955–964. 10.1093/nar/25.5.955 9023104 PMC146525

[B34] Ma X. Huang B. (2016). Gibberellin-stimulation of rhizome elongation and differential GA-responsive proteomic changes in two grass species. Front. In Plant Sci. 7, 905. 10.3389/fpls.2016.00905 27446135 PMC4917561

[B35] Meyer W. A. Funk C. R. (1989). Progress and Benefits to Humanity from Breeding Cool-Season Grasses for Turf 1. Contributions from breeding forage and turf grasses, 31-48.

[B36] Piper C. V. (1906). North American species of Festuca. Department of Botany, Smithsonian Institution: US Government Printing Office. 10.5962/bhl.title.53679

[B37] Provan J. Powell W. Hollingsworth P. M. (2001). Chloroplast microsatellites: new tools for studies in plant ecology and evolution. Trends In Ecol. Evol. 16, 142–147. 10.1016/S0169-5347(00)02097-8 11179578

[B38] Qiu Y. Hirsch C. D. Yang Y. Watkins E. (2019). Towards improved molecular identification tools in fine fescue (Festuca L., poaceae) turfgrasses: nuclear genome size, ploidy, and chloroplast genome sequencing. bioRxiv, 708149. 10.1101/708149. PMC6909427 31867041

[B39] Quinlan A. R. Hall I. M. (2010). BEDTools: a flexible suite of utilities for comparing genomic features. Bioinform. 26, 841–842. 10.1093/bioinformatics/btq033 PMC2832824 20110278

[B40] Rambaut A. (2012). FigTree v1. 4.

[B41] Robbins M. D. Staub J. E. Bushman B. S. (2016). Development of fine-leaved Festuca grass populations identifies genetic resources having improved forage production with potential for wildfire control in the western United States. Euphytica 209, 377–393. 10.1007/s10681-016-1644-z

[B42] Rousseau-Gueutin M. Huang X. Higginson E. Ayliffe M. Day A. Timmis J. N. (2013). Potential functional replacement of the plastidic acetyl-CoA carboxylase subunit (accD) gene by recent transfers to the nucleus in some angiosperm lineages. Plant Physiol. 161, 1918–1929. 10.1104/pp.113.214528 23435694 PMC3613465

[B43] Ruemmele B. Brilman L. Huff D. (1995). Fine fescue germplasm diversity and vulnerability. Crop Sci. 35, 313–316. 10.2135/cropsci1995.0011183X003500020003x

[B44] Stamatakis A. (2006). RAxML-VI-HPC: maximum likelihood-based phylogenetic analyses with thousands of taxa and mixed models. Bioinform. 22, 2688–2690. 10.1093/bioinformatics/btl446 16928733

[B45] Thiel T. Michalek W. Varshney R. Graner A. (2003). Exploiting EST databases for the development and characterization of gene-derived SSR-markers in barley (Hordeum vulgare L.). Theor. Appl. Genet. 106, 411–422. 10.1007/s00122-002-1031-0 12589540

[B46] Tillich M. Lehwark P. Pellizzer T. Ulbricht-Jones E. S. Fischer A. Bock R. (2017). GeSeq-versatile and accurate annotation of organelle genomes. Nucleic Acids Res. 45, W6–W11. 10.1093/nar/gkx391 28486635 PMC5570176

[B47] Torrecilla P. Catalán P. (2002). Phylogeny of broad-leaved and fine-leaved Festuca lineages (Poaceae) based on nuclear ITS sequences. Syst. Bot. 27, 241–252. 10.1043/0363-6445-27.2.241

[B48] Untergasser A. Cutcutache I. Koressaar T. Ye J. Faircloth B. C. Remm M. (2012). Primer3-new capabilities and interfaces. Nucleic Acids Res. 40, e115–e115. 10.1093/nar/gks596 22730293 PMC3424584

[B49] Wyman S. K. Jansen R. K. Boore J. L. (2004). Automatic annotation of organellar genomes with DOGMA. Bioinform. 20, 3252–3255. 10.1093/bioinformatics/bth352 15180927

[B50] Yang M. Zhu L. Pan C. Xu L. Liu Y. Ke W. (2015). Transcriptomic analysis of the regulation of rhizome formation in temperate and tropical lotus (Nelumbo nucifera). Sci. Rep. 5, 13059. 10.1038/srep13059 26279185 PMC4538393

