# Supplementary material for: Towards Improved Molecular Identification Tools in Fine Fescue (Festuca L., Poaceae) Turfgrasses: Nuclear Genome Size, Ploidy, and Chloroplast Genome Sequencing
Source: Front Genet. 2019 Dec 6;10:1223. doi: 10.3389/fgene.2019.01223 (PMC6909427; doi:10.3389/fgene.2019.01223)
Supplement: Supplementary file 2 [file Table_2.docx]

Table S2. SSR loci types and number distributions of fine fescue species predicted using MISA program.

| Repeats | SSR | Cultivar name | Taxon |
| --- | --- | --- | --- |
| A/T | 24 | Shoreline | *F. rubra* ssp. *litoralis* |
| C/G | 1 | Shoreline | *F. rubra* ssp. *litoralis* |
| AG/CT | 1 | Shoreline | *F. rubra* ssp. *litoralis* |
| AT/AT | 6 | Shoreline | *F. rubra* ssp. *litoralis* |
| AAC/GTT | 1 | Shoreline | *F. rubra* ssp. *litoralis* |
| AAG/CTT | 1 | Shoreline | *F. rubra* ssp. *litoralis* |
| AAT/ATT | 1 | Shoreline | *F. rubra* ssp. *litoralis* |
| AAAC/GTTT | 1 | Shoreline | *F. rubra* ssp. *litoralis* |
| AAAG/CTTT | 2 | Shoreline | *F. rubra* ssp. *litoralis* |
| AAAT/ATTT | 1 | Shoreline | *F. rubra* ssp. *litoralis* |
| AACG/CGTT | 2 | Shoreline | *F. rubra* ssp. *litoralis* |
| AAGG/CCTT | 1 | Shoreline | *F. rubra* ssp. *litoralis* |
| AATG/ATTC | 1 | Shoreline | *F. rubra* ssp. *litoralis* |
| ACAT/ATGT | 1 | Shoreline | *F. rubra* ssp. *litoralis* |
| AAATT/AATTT | 1 | Shoreline | *F. rubra* ssp. *litoralis* |
| AATGC/ATTGC | 1 | Shoreline | *F. rubra* ssp. *litoralis* |
| A/T | 23 | Quatro | *F. ovina* |
| C/G | 1 | Quatro | *F. ovina* |
| AG/CT | 1 | Quatro | *F. ovina* |
| AT/AT | 6 | Quatro | *F. ovina* |
| AAC/GTT | 1 | Quatro | *F. ovina* |
| AAG/CTT | 2 | Quatro | *F. ovina* |
| AAT/ATT | 1 | Quatro | *F. ovina* |
| AAAC/GTTT | 1 | Quatro | *F. ovina* |
| AAAG/CTTT | 1 | Quatro | *F. ovina* |
| AAAT/ATTT | 1 | Quatro | *F. ovina* |
| AACG/CGTT | 2 | Quatro | *F. ovina* |
| AAGG/CCTT | 1 | Quatro | *F. ovina* |
| AATG/ATTC | 1 | Quatro | *F. ovina* |
| ACAT/ATGT | 1 | Quatro | *F. ovina* |
| AATGC/ATTGC | 1 | Quatro | *F. ovina* |
| ACCAT/ATGGT | 1 | Quatro | *F. ovina* |
| A/T | 18 | Beacon | *F. brevipila* |
| C/G | 1 | Beacon | *F. brevipila* |
| AG/CT | 1 | Beacon | *F. brevipila* |
| AT/AT | 5 | Beacon | *F. brevipila* |
| AAC/GTT | 1 | Beacon | *F. brevipila* |
| AAG/CTT | 2 | Beacon | *F. brevipila* |
| AAT/ATT | 1 | Beacon | *F. brevipila* |
| AAAC/GTTT | 1 | Beacon | *F. brevipila* |
| AAAG/CTTT | 1 | Beacon | *F. brevipila* |
| AAAT/ATTT | 1 | Beacon | *F. brevipila* |
| AACG/CGTT | 2 | Beacon | *F. brevipila* |
| AAGG/CCTT | 1 | Beacon | *F. brevipila* |
| AATG/ATTC | 1 | Beacon | *F. brevipila* |
| ACAT/ATGT | 1 | Beacon | *F. brevipila* |
| AATGC/ATTGC | 1 | Beacon | *F. brevipila* |
| ACCAT/ATGGT | 1 | Beacon | *F. brevipila* |
| A/T | 22 | Navigator II | *F. rubra* ssp. *rubra* |
| C/G | 1 | Navigator II | *F. rubra* ssp. *rubra* |
| AG/CT | 1 | Navigator II | *F. rubra* ssp. *rubra* |
| AT/AT | 6 | Navigator II | *F. rubra* ssp. *rubra* |
| AAC/GTT | 1 | Navigator II | *F. rubra* ssp. *rubra* |
| AAG/CTT | 2 | Navigator II | *F. rubra* ssp. *rubra* |
| AAT/ATT | 1 | Navigator II | *F. rubra* ssp. *rubra* |
| AAAC/GTTT | 1 | Navigator II | *F. rubra* ssp. *rubra* |
| AAAG/CTTT | 2 | Navigator II | *F. rubra* ssp. *rubra* |
| AAAT/ATTT | 1 | Navigator II | *F. rubra* ssp. *rubra* |
| AACG/CGTT | 2 | Navigator II | *F. rubra* ssp. *rubra* |
| AAGG/CCTT | 1 | Navigator II | *F. rubra* ssp. *rubra* |
| AATG/ATTC | 1 | Navigator II | *F. rubra* ssp. *rubra* |
| ACAT/ATGT | 1 | Navigator II | *F. rubra* ssp. *rubra* |
| AAATT/AATTT | 1 | Navigator II | *F. rubra* ssp. *rubra* |
| AATGC/ATTGC | 1 | Navigator II | *F. rubra* ssp. *rubra* |
| A/T | 20 | Treazure II | *F. rubra* ssp. *fallax* |
| C/G | 1 | Treazure II | *F. rubra* ssp. *fallax* |
| AG/CT | 1 | Treazure II | *F. rubra* ssp. *fallax* |
| AT/AT | 6 | Treazure II | *F. rubra* ssp. *fallax* |
| AAC/GTT | 1 | Treazure II | *F. rubra* ssp. *fallax* |
| AAG/CTT | 2 | Treazure II | *F. rubra* ssp. *fallax* |
| AAT/ATT | 1 | Treazure II | *F. rubra* ssp. *fallax* |
| AAAC/GTTT | 1 | Treazure II | *F. rubra* ssp. *fallax* |
| AAAG/CTTT | 2 | Treazure II | *F. rubra* ssp. *fallax* |
| AAAT/ATTT | 1 | Treazure II | *F. rubra* ssp. *fallax* |
| AACG/CGTT | 2 | Treazure II | *F. rubra* ssp. *fallax* |
| AAGG/CCTT | 1 | Treazure II | *F. rubra* ssp. *fallax* |
| AATG/ATTC | 1 | Treazure II | *F. rubra* ssp. *fallax* |
| ACAT/ATGT | 1 | Treazure II | *F. rubra* ssp. *fallax* |
| AATGC/ATTGC | 1 | Treazure II | *F. rubra* ssp. *fallax* |
| AAATT/AATTT | 0 | Beacon | *F. brevipila* |
| AAATT/AATTT | 0 | Quatro | *F. ovina* |
| AAATT/AATTT | 0 | Treazure II | *F. rubra* ssp. *fallax* |
| ACCAT/ATGGT | 0 | Treazure II | *F. rubra* ssp. *fallax* |
| ACCAT/ATGGT | 0 | Shoreline | *F. rubra* ssp. *litoralis* |
| ACCAT/ATGGT | 0 | Navigator II | *F. rubra* ssp. *rubra* |
