# Supplementary material for: Towards Improved Molecular Identification Tools in Fine Fescue (Festuca L., Poaceae) Turfgrasses: Nuclear Genome Size, Ploidy, and Chloroplast Genome Sequencing
Source: Front Genet. 2019 Dec 6;10:1223. doi: 10.3389/fgene.2019.01223 (PMC6909427; doi:10.3389/fgene.2019.01223)
Supplement: Supplementary file 6 [file Table_6.docx]

**Table S6.** Sliding window analysis for average nucleotide diversity calculation

the fine fescue chloroplast genomes

|  |  |  |
| --- | --- | --- |
| Midpoint | Pi | Position |
| 306 | 0.002 | 0.306 |
| 506 | 0 | 0.506 |
| 706 | 0 | 0.706 |
| 906 | 0 | 0.906 |
| 1106 | 0.002 | 1.106 |
| 1306 | 0.00367 | 1.306 |
| 1506 | 0.00633 | 1.506 |
| 1713 | 0.00533 | 1.713 |
| 1913 | 0.00533 | 1.913 |
| 2113 | 0.00267 | 2.113 |
| 2313 | 0.00367 | 2.313 |
| 2513 | 0.003 | 2.513 |
| 2713 | 0.003 | 2.713 |
| 2913 | 0.001 | 2.913 |
| 3113 | 0.001 | 3.113 |
| 3313 | 0.00233 | 3.313 |
| 3513 | 0.003 | 3.513 |
| 3714 | 0.002 | 3.714 |
| 3922 | 0.00767 | 3.922 |
| 4122 | 0.00767 | 4.122 |
| 4322 | 0.00867 | 4.322 |
| 4526 | 0.00333 | 4.526 |
| 4726 | 0.00567 | 4.726 |
| 4927 | 0.00733 | 4.927 |
| 5127 | 0.007 | 5.127 |
| 5327 | 0.005 | 5.327 |
| 5527 | 0.005 | 5.527 |
| 5727 | 0.009 | 5.727 |
| 5927 | 0.012 | 5.927 |
| 6329 | 0.011 | 6.329 |
| 6529 | 0.00667 | 6.529 |
| 6729 | 0.00267 | 6.729 |
| 6929 | 0.00867 | 6.929 |
| 7129 | 0.01167 | 7.129 |
| 7335 | 0.01167 | 7.335 |
| 7536 | 0.006 | 7.536 |
| 7736 | 0.00767 | 7.736 |
| 7937 | 0.00867 | 7.937 |
| 8150 | 0.00833 | 8.15 |
| 8356 | 0.00767 | 8.356 |
| 8557 | 0.00667 | 8.557 |
| 8757 | 0.00567 | 8.757 |
| 8957 | 0.00267 | 8.957 |
| 9157 | 0.00367 | 9.157 |
| 9357 | 0.00367 | 9.357 |
| 9557 | 0.00167 | 9.557 |
| 9757 | 0.00067 | 9.757 |
| 9957 | 0 | 9.957 |
| 10157 | 0.002 | 10.157 |
| 10357 | 0.002 | 10.357 |
| 10557 | 0.003 | 10.557 |
| 10757 | 0.002 | 10.757 |
| 10957 | 0.002 | 10.957 |
| 11157 | 0.003 | 11.157 |
| 11357 | 0.002 | 11.357 |
| 11557 | 0.00267 | 11.557 |
| 11757 | 0.00267 | 11.757 |
| 11957 | 0.00267 | 11.957 |
| 12157 | 0.002 | 12.157 |
| 12357 | 0 | 12.357 |
| 12717 | 0.00167 | 12.717 |
| 12922 | 0.00267 | 12.922 |
| 13122 | 0.00367 | 13.122 |
| 13322 | 0.002 | 13.322 |
| 13522 | 0.003 | 13.522 |
| 13722 | 0.00567 | 13.722 |
| 13922 | 0.01567 | 13.922 |
| 14129 | 0.01533 | 14.129 |
| 14331 | 0.01467 | 14.331 |
| 14531 | 0.00567 | 14.531 |
| 14734 | 0.004 | 14.734 |
| 14934 | 0.00267 | 14.934 |
| 15134 | 0.00233 | 15.134 |
| 15342 | 0.00233 | 15.342 |
| 15563 | 0.002 | 15.563 |
| 15991 | 0.00133 | 15.991 |
| 16192 | 0.00133 | 16.192 |
| 16392 | 0.002 | 16.392 |
| 16598 | 0.00367 | 16.598 |
| 16798 | 0.00667 | 16.798 |
| 16998 | 0.00867 | 16.998 |
| 17205 | 0.00967 | 17.205 |
| 17409 | 0.01067 | 17.409 |
| 17609 | 0.01067 | 17.609 |
| 17809 | 0.01033 | 17.809 |
| 18009 | 0.008 | 18.009 |
| 18214 | 0.00733 | 18.214 |
| 18415 | 0.008 | 18.415 |
| 18615 | 0.00733 | 18.615 |
| 18815 | 0.004 | 18.815 |
| 19015 | 0.001 | 19.015 |
| 19215 | 0 | 19.215 |
| 19415 | 0 | 19.415 |
| 19615 | 0 | 19.615 |
| 19815 | 0.00067 | 19.815 |
| 20015 | 0.00067 | 20.015 |
| 20215 | 0.00167 | 20.215 |
| 20415 | 0.003 | 20.415 |
| 20615 | 0.00667 | 20.615 |
| 20815 | 0.00633 | 20.815 |
| 21015 | 0.00733 | 21.015 |
| 21215 | 0.00467 | 21.215 |
| 21415 | 0.006 | 21.415 |
| 21615 | 0.003 | 21.615 |
| 21815 | 0.002 | 21.815 |
| 22015 | 0.00067 | 22.015 |
| 22215 | 0.00067 | 22.215 |
| 22415 | 0.00067 | 22.415 |
| 22615 | 0.00167 | 22.615 |
| 22815 | 0.00167 | 22.815 |
| 23015 | 0.00167 | 23.015 |
| 23215 | 0 | 23.215 |
| 23415 | 0.00067 | 23.415 |
| 23615 | 0.00167 | 23.615 |
| 23815 | 0.00167 | 23.815 |
| 24015 | 0.00233 | 24.015 |
| 24215 | 0.00333 | 24.215 |
| 24415 | 0.005 | 24.415 |
| 24615 | 0.00467 | 24.615 |
| 24815 | 0.00267 | 24.815 |
| 25015 | 0.001 | 25.015 |
| 25215 | 0.001 | 25.215 |
| 25415 | 0.00367 | 25.415 |
| 25615 | 0.00533 | 25.615 |
| 25815 | 0.009 | 25.815 |
| 26015 | 0.00733 | 26.015 |
| 26215 | 0.00867 | 26.215 |
| 26436 | 0.00467 | 26.436 |
| 26636 | 0.00367 | 26.636 |
| 26836 | 0.00433 | 26.836 |
| 27036 | 0.00467 | 27.036 |
| 27236 | 0.00867 | 27.236 |
| 27436 | 0.005 | 27.436 |
| 27636 | 0.009 | 27.636 |
| 27836 | 0.005 | 27.836 |
| 28036 | 0.005 | 28.036 |
| 28236 | 0 | 28.236 |
| 28436 | 0 | 28.436 |
| 28636 | 0.00267 | 28.636 |
| 28836 | 0.00267 | 28.836 |
| 29047 | 0.00267 | 29.047 |
| 29251 | 0 | 29.251 |
| 29451 | 0.001 | 29.451 |
| 29651 | 0.00167 | 29.651 |
| 29851 | 0.00267 | 29.851 |
| 30057 | 0.00167 | 30.057 |
| 30257 | 0.002 | 30.257 |
| 30457 | 0.001 | 30.457 |
| 30657 | 0.00367 | 30.657 |
| 30857 | 0.00467 | 30.857 |
| 31059 | 0.00567 | 31.059 |
| 31262 | 0.004 | 31.262 |
| 31463 | 0.00267 | 31.463 |
| 31663 | 0.00267 | 31.663 |
| 31864 | 0.00167 | 31.864 |
| 32064 | 0.002 | 32.064 |
| 32264 | 0.00167 | 32.264 |
| 32464 | 0.00567 | 32.464 |
| 32667 | 0.00833 | 32.667 |
| 32867 | 0.00767 | 32.867 |
| 33067 | 0.00633 | 33.067 |
| 33267 | 0.00333 | 33.267 |
| 33468 | 0.00433 | 33.468 |
| 33668 | 0.00467 | 33.668 |
| 33868 | 0.004 | 33.868 |
| 34068 | 0.003 | 34.068 |
| 34268 | 0.001 | 34.268 |
| 34468 | 0.001 | 34.468 |
| 34668 | 0.002 | 34.668 |
| 34868 | 0.00167 | 34.868 |
| 35068 | 0.00433 | 35.068 |
| 35268 | 0.005 | 35.268 |
| 35468 | 0.00533 | 35.468 |
| 35668 | 0.00367 | 35.668 |
| 35868 | 0.002 | 35.868 |
| 36068 | 0.002 | 36.068 |
| 36268 | 0.004 | 36.268 |
| 36468 | 0.00467 | 36.468 |
| 36668 | 0.00367 | 36.668 |
| 36868 | 0.00067 | 36.868 |
| 37068 | 0.001 | 37.068 |
| 37268 | 0.003 | 37.268 |
| 37468 | 0.003 | 37.468 |
| 37668 | 0.002 | 37.668 |
| 37868 | 0 | 37.868 |
| 38068 | 0.001 | 38.068 |
| 38268 | 0.001 | 38.268 |
| 38468 | 0.001 | 38.468 |
| 38668 | 0.002 | 38.668 |
| 38868 | 0.002 | 38.868 |
| 39068 | 0.003 | 39.068 |
| 39268 | 0.00267 | 39.268 |
| 39468 | 0.00267 | 39.468 |
| 39668 | 0.00167 | 39.668 |
| 39868 | 0.001 | 39.868 |
| 40068 | 0.00167 | 40.068 |
| 40268 | 0.00167 | 40.268 |
| 40468 | 0.00133 | 40.468 |
| 40669 | 0.00333 | 40.669 |
| 40876 | 0.00833 | 40.876 |
| 41086 | 0.01 | 41.086 |
| 41286 | 0.008 | 41.286 |
| 41486 | 0.003 | 41.486 |
| 41690 | 0.00167 | 41.69 |
| 41890 | 0.002 | 41.89 |
| 42093 | 0.002 | 42.093 |
| 42293 | 0.002 | 42.293 |
| 42493 | 0.001 | 42.493 |
| 42693 | 0.00267 | 42.693 |
| 42893 | 0.00167 | 42.893 |
| 43097 | 0.004 | 43.097 |
| 43297 | 0.004 | 43.297 |
| 43509 | 0.005 | 43.509 |
| 43716 | 0.00633 | 43.716 |
| 43932 | 0.00533 | 43.932 |
| 44134 | 0.00433 | 44.134 |
| 44334 | 0.00067 | 44.334 |
| 44534 | 0.00067 | 44.534 |
| 44734 | 0.00433 | 44.734 |
| 44943 | 0.00833 | 44.943 |
| 45145 | 0.01233 | 45.145 |
| 45348 | 0.01133 | 45.348 |
| 45558 | 0.015 | 45.558 |
| 45764 | 0.01133 | 45.764 |
| 45969 | 0.01133 | 45.969 |
| 46169 | 0.007 | 46.169 |
| 46373 | 0.007 | 46.373 |
| 46573 | 0.01433 | 46.573 |
| 46781 | 0.012 | 46.781 |
| 46999 | 0.01633 | 46.999 |
| 47209 | 0.00633 | 47.209 |
| 47409 | 0.00733 | 47.409 |
| 47613 | 0.00267 | 47.613 |
| 47813 | 0.00467 | 47.813 |
| 48013 | 0.00267 | 48.013 |
| 48219 | 0.004 | 48.219 |
| 48419 | 0.004 | 48.419 |
| 48619 | 0.005 | 48.619 |
| 48819 | 0.004 | 48.819 |
| 49019 | 0.002 | 49.019 |
| 49219 | 0.002 | 49.219 |
| 49419 | 0.00167 | 49.419 |
| 49644 | 0.00333 | 49.644 |
| 49914 | 0.00467 | 49.914 |
| 50114 | 0.006 | 50.114 |
| 50315 | 0.00433 | 50.315 |
| 50515 | 0.006 | 50.515 |
| 50715 | 0.005 | 50.715 |
| 50915 | 0.005 | 50.915 |
| 51115 | 0.002 | 51.115 |
| 51315 | 0.002 | 51.315 |
| 51515 | 0.004 | 51.515 |
| 51715 | 0.004 | 51.715 |
| 51915 | 0.00467 | 51.915 |
| 52115 | 0.00433 | 52.115 |
| 52315 | 0.00433 | 52.315 |
| 52515 | 0.00467 | 52.515 |
| 52715 | 0.005 | 52.715 |
| 52915 | 0.004 | 52.915 |
| 53115 | 0.002 | 53.115 |
| 53316 | 0.001 | 53.316 |
| 53518 | 0.008 | 53.518 |
| 53727 | 0.011 | 53.727 |
| 53932 | 0.012 | 53.932 |
| 54132 | 0.007 | 54.132 |
| 54332 | 0.008 | 54.332 |
| 54532 | 0.00667 | 54.532 |
| 54732 | 0.00667 | 54.732 |
| 54932 | 0.00467 | 54.932 |
| 55132 | 0.00567 | 55.132 |
| 55332 | 0.00467 | 55.332 |
| 55562 | 0.00367 | 55.562 |
| 55762 | 0.006 | 55.762 |
| 56237 | 0.00567 | 56.237 |
| 56439 | 0.00567 | 56.439 |
| 56639 | 0.00367 | 56.639 |
| 56844 | 0.003 | 56.844 |
| 57045 | 0.002 | 57.045 |
| 57245 | 0.00267 | 57.245 |
| 57445 | 0.00267 | 57.445 |
| 57857 | 0.004 | 57.857 |
| 58057 | 0.002 | 58.057 |
| 58257 | 0.002 | 58.257 |
| 58457 | 0.00133 | 58.457 |
| 58657 | 0.00067 | 58.657 |
| 58859 | 0.00067 | 58.859 |
| 59060 | 0 | 59.06 |
| 59260 | 0.001 | 59.26 |
| 59460 | 0.00167 | 59.46 |
| 59660 | 0.00367 | 59.66 |
| 59860 | 0.00467 | 59.86 |
| 60060 | 0.00567 | 60.06 |
| 60263 | 0.00733 | 60.263 |
| 60463 | 0.00633 | 60.463 |
| 60663 | 0.00733 | 60.663 |
| 60863 | 0.00367 | 60.863 |
| 61063 | 0.00267 | 61.063 |
| 61263 | 0 | 61.263 |
| 61463 | 0.00367 | 61.463 |
| 61663 | 0.00633 | 61.663 |
| 61868 | 0.009 | 61.868 |
| 62085 | 0.00633 | 62.085 |
| 62375 | 0.00533 | 62.375 |
| 62576 | 0.00333 | 62.576 |
| 62776 | 0.004 | 62.776 |
| 62976 | 0.00333 | 62.976 |
| 63176 | 0.00467 | 63.176 |
| 63382 | 0.005 | 63.382 |
| 63582 | 0.004 | 63.582 |
| 63782 | 0.003 | 63.782 |
| 64051 | 0.003 | 64.051 |
| 64258 | 0.003 | 64.258 |
| 64458 | 0.002 | 64.458 |
| 64663 | 0.002 | 64.663 |
| 64866 | 0.002 | 64.866 |
| 65066 | 0.002 | 65.066 |
| 65266 | 0.00133 | 65.266 |
| 65466 | 0.00133 | 65.466 |
| 65666 | 0.00133 | 65.666 |
| 65866 | 0 | 65.866 |
| 66067 | 0 | 66.067 |
| 66267 | 0.003 | 66.267 |
| 66467 | 0.003 | 66.467 |
| 66668 | 0.00667 | 66.668 |
| 66875 | 0.00367 | 66.875 |
| 67075 | 0.00567 | 67.075 |
| 67275 | 0.003 | 67.275 |
| 67475 | 0.007 | 67.475 |
| 67678 | 0.006 | 67.678 |
| 67878 | 0.006 | 67.878 |
| 68078 | 0.003 | 68.078 |
| 68278 | 0.003 | 68.278 |
| 68478 | 0.002 | 68.478 |
| 68678 | 0.002 | 68.678 |
| 68878 | 0.002 | 68.878 |
| 69078 | 0.003 | 69.078 |
| 69278 | 0.002 | 69.278 |
| 69478 | 0.002 | 69.478 |
| 69678 | 0.00167 | 69.678 |
| 69878 | 0.00267 | 69.878 |
| 70078 | 0.00167 | 70.078 |
| 70278 | 0.001 | 70.278 |
| 70478 | 0.001 | 70.478 |
| 70678 | 0.00267 | 70.678 |
| 70879 | 0.00433 | 70.879 |
| 71080 | 0.00333 | 71.08 |
| 71280 | 0.00367 | 71.28 |
| 71480 | 0.002 | 71.48 |
| 71680 | 0.006 | 71.68 |
| 71880 | 0.00567 | 71.88 |
| 72080 | 0.00667 | 72.08 |
| 72280 | 0.00433 | 72.28 |
| 72480 | 0.00267 | 72.48 |
| 72680 | 0.00333 | 72.68 |
| 72880 | 0.00167 | 72.88 |
| 73080 | 0.00167 | 73.08 |
| 73280 | 0.00267 | 73.28 |
| 73488 | 0.00367 | 73.488 |
| 73688 | 0.00467 | 73.688 |
| 73888 | 0.00333 | 73.888 |
| 74088 | 0.006 | 74.088 |
| 74288 | 0.007 | 74.288 |
| 74494 | 0.00567 | 74.494 |
| 74694 | 0.002 | 74.694 |
| 74894 | 0.00367 | 74.894 |
| 75095 | 0.00367 | 75.095 |
| 75296 | 0.00567 | 75.296 |
| 75496 | 0.00533 | 75.496 |
| 75696 | 0.00533 | 75.696 |
| 75896 | 0.00333 | 75.896 |
| 76096 | 0.002 | 76.096 |
| 76299 | 0.004 | 76.299 |
| 76509 | 0.005 | 76.509 |
| 76709 | 0.00467 | 76.709 |
| 76910 | 0.00367 | 76.91 |
| 77110 | 0.00367 | 77.11 |
| 77310 | 0.003 | 77.31 |
| 77514 | 0.006 | 77.514 |
| 77714 | 0.005 | 77.714 |
| 77914 | 0.00567 | 77.914 |
| 78114 | 0.00367 | 78.114 |
| 78324 | 0.00367 | 78.324 |
| 78524 | 0.003 | 78.524 |
| 78724 | 0.003 | 78.724 |
| 78924 | 0.00567 | 78.924 |
| 79124 | 0.00467 | 79.124 |
| 79324 | 0.00467 | 79.324 |
| 79524 | 0.00867 | 79.524 |
| 79725 | 0.00867 | 79.725 |
| 79925 | 0.00767 | 79.925 |
| 80125 | 0.001 | 80.125 |
| 80325 | 0.001 | 80.325 |
| 80525 | 0 | 80.525 |
| 80725 | 0 | 80.725 |
| 80925 | 0 | 80.925 |
| 81125 | 0.001 | 81.125 |
| 81325 | 0.001 | 81.325 |
| 81525 | 0.001 | 81.525 |
| 81725 | 0 | 81.725 |
| 81925 | 0.001 | 81.925 |
| 82125 | 0.003 | 82.125 |
| 82325 | 0.00367 | 82.325 |
| 82525 | 0.00333 | 82.525 |
| 82725 | 0.002 | 82.725 |
| 82925 | 0.00233 | 82.925 |
| 83125 | 0.00167 | 83.125 |
| 83325 | 0.002 | 83.325 |
| 83525 | 0.001 | 83.525 |
| 83725 | 0.001 | 83.725 |
| 83925 | 0 | 83.925 |
| 84125 | 0 | 84.125 |
| 84325 | 0 | 84.325 |
| 84525 | 0 | 84.525 |
| 84725 | 0 | 84.725 |
| 84925 | 0.001 | 84.925 |
| 85125 | 0.001 | 85.125 |
| 85325 | 0.001 | 85.325 |
| 85525 | 0 | 85.525 |
| 85725 | 0 | 85.725 |
| 85925 | 0 | 85.925 |
| 86125 | 0 | 86.125 |
| 86325 | 0 | 86.325 |
| 86525 | 0 | 86.525 |
| 86725 | 0 | 86.725 |
| 86925 | 0 | 86.925 |
| 87125 | 0 | 87.125 |
| 87325 | 0 | 87.325 |
| 87525 | 0 | 87.525 |
| 87725 | 0 | 87.725 |
| 87925 | 0 | 87.925 |
| 88125 | 0 | 88.125 |
| 88325 | 0 | 88.325 |
| 88525 | 0 | 88.525 |
| 88725 | 0 | 88.725 |
| 88925 | 0 | 88.925 |
| 89125 | 0.00067 | 89.125 |
| 89325 | 0.00067 | 89.325 |
| 89525 | 0.00167 | 89.525 |
| 89725 | 0.001 | 89.725 |
| 89925 | 0.002 | 89.925 |
| 90144 | 0.002 | 90.144 |
| 90344 | 0.002 | 90.344 |
| 90544 | 0.001 | 90.544 |
| 90744 | 0 | 90.744 |
| 90944 | 0 | 90.944 |
| 91144 | 0 | 91.144 |
| 91344 | 0 | 91.344 |
| 91544 | 0 | 91.544 |
| 91744 | 0 | 91.744 |
| 91944 | 0 | 91.944 |
| 92144 | 0 | 92.144 |
| 92344 | 0.001 | 92.344 |
| 92544 | 0.001 | 92.544 |
| 92744 | 0.001 | 92.744 |
| 92944 | 0.00067 | 92.944 |
| 93144 | 0.00167 | 93.144 |
| 93344 | 0.00167 | 93.344 |
| 93544 | 0.001 | 93.544 |
| 93744 | 0 | 93.744 |
| 93944 | 0 | 93.944 |
| 94144 | 0 | 94.144 |
| 94344 | 0.001 | 94.344 |
| 94544 | 0.001 | 94.544 |
| 94744 | 0.001 | 94.744 |
| 94944 | 0 | 94.944 |
| 95144 | 0 | 95.144 |
| 95344 | 0 | 95.344 |
| 95544 | 0 | 95.544 |
| 95744 | 0 | 95.744 |
| 95944 | 0 | 95.944 |
| 96144 | 0 | 96.144 |
| 96344 | 0 | 96.344 |
| 96544 | 0 | 96.544 |
| 96744 | 0 | 96.744 |
| 96944 | 0 | 96.944 |
| 97144 | 0 | 97.144 |
| 97344 | 0 | 97.344 |
| 97544 | 0 | 97.544 |
| 97744 | 0 | 97.744 |
| 97944 | 0 | 97.944 |
| 98144 | 0 | 98.144 |
| 98344 | 0 | 98.344 |
| 98544 | 0.002 | 98.544 |
| 98744 | 0.002 | 98.744 |
| 98944 | 0.002 | 98.944 |
| 99144 | 0 | 99.144 |
| 99344 | 0 | 99.344 |
| 99544 | 0.001 | 99.544 |
| 99744 | 0.001 | 99.744 |
| 99944 | 0.00367 | 99.944 |
| 100145 | 0.00267 | 100.145 |
| 100345 | 0.00267 | 100.345 |
| 100545 | 0 | 100.545 |
| 100746 | 0.002 | 100.746 |
| 100956 | 0.00367 | 100.956 |
| 101156 | 0.00767 | 101.156 |
| 101356 | 0.00733 | 101.356 |
| 101556 | 0.00667 | 101.556 |
| 101756 | 0.00333 | 101.756 |
| 101956 | 0.00167 | 101.956 |
| 102156 | 0.00333 | 102.156 |
| 102356 | 0.00267 | 102.356 |
| 102556 | 0.00267 | 102.556 |
| 102756 | 0.001 | 102.756 |
| 102956 | 0.00267 | 102.956 |
| 103156 | 0.007 | 103.156 |
| 103361 | 0.00967 | 103.361 |
| 103566 | 0.01233 | 103.566 |
| 103768 | 0.00867 | 103.768 |
| 103985 | 0.008 | 103.985 |
| 104185 | 0.01333 | 104.185 |
| 104385 | 0.01767 | 104.385 |
| 104614 | 0.01633 | 104.614 |
| 104820 | 0.01167 | 104.82 |
| 105020 | 0.00667 | 105.02 |
| 105220 | 0.00967 | 105.22 |
| 105420 | 0.00667 | 105.42 |
| 105620 | 0.00933 | 105.62 |
| 105820 | 0.00633 | 105.82 |
| 106026 | 0.00433 | 106.026 |
| 106226 | 0.00167 | 106.226 |
| 106426 | 0.001 | 106.426 |
| 106626 | 0.002 | 106.626 |
| 106826 | 0.00267 | 106.826 |
| 107026 | 0.00167 | 107.026 |
| 107226 | 0.00167 | 107.226 |
| 107427 | 0.001 | 107.427 |
| 107627 | 0.004 | 107.627 |
| 107827 | 0.00367 | 107.827 |
| 108027 | 0.00533 | 108.027 |
| 108227 | 0.00333 | 108.227 |
| 108427 | 0.00433 | 108.427 |
| 108627 | 0.00833 | 108.627 |
| 108827 | 0.008 | 108.827 |
| 109027 | 0.00733 | 109.027 |
| 109227 | 0.00267 | 109.227 |
| 109427 | 0.004 | 109.427 |
| 109628 | 0.003 | 109.628 |
| 109828 | 0.004 | 109.828 |
| 110028 | 0.003 | 110.028 |
| 110232 | 0.003 | 110.232 |
| 110432 | 0.003 | 110.432 |
| 110632 | 0.005 | 110.632 |
| 110832 | 0.006 | 110.832 |
| 111037 | 0.007 | 111.037 |
| 111245 | 0.004 | 111.245 |
| 111445 | 0.005 | 111.445 |
| 111646 | 0.003 | 111.646 |
| 111852 | 0.00667 | 111.852 |
| 112052 | 0.00467 | 112.052 |
| 112252 | 0.00467 | 112.252 |
| 112452 | 0.002 | 112.452 |
| 112652 | 0.00267 | 112.652 |
| 112852 | 0.00467 | 112.852 |
| 113052 | 0.00567 | 113.052 |
| 113252 | 0.005 | 113.252 |
| 113452 | 0.002 | 113.452 |
| 113653 | 0 | 113.653 |
| 113853 | 0.00167 | 113.853 |
| 114053 | 0.00267 | 114.053 |
| 114254 | 0.00367 | 114.254 |
| 114454 | 0.002 | 114.454 |
| 114654 | 0.001 | 114.654 |
| 114854 | 0 | 114.854 |
| 115054 | 0 | 115.054 |
| 115254 | 0.002 | 115.254 |
| 115454 | 0.002 | 115.454 |
| 115654 | 0.002 | 115.654 |
| 115854 | 0 | 115.854 |
| 116054 | 0 | 116.054 |
| 116254 | 0 | 116.254 |
| 116454 | 0 | 116.454 |
| 116654 | 0 | 116.654 |
| 116854 | 0 | 116.854 |
| 117054 | 0 | 117.054 |
| 117254 | 0 | 117.254 |
| 117454 | 0 | 117.454 |
| 117654 | 0 | 117.654 |
| 117854 | 0 | 117.854 |
| 118054 | 0 | 118.054 |
| 118254 | 0 | 118.254 |
| 118454 | 0 | 118.454 |
| 118654 | 0 | 118.654 |
| 118854 | 0 | 118.854 |
| 119054 | 0 | 119.054 |
| 119254 | 0 | 119.254 |
| 119454 | 0.001 | 119.454 |
| 119654 | 0.001 | 119.654 |
| 119854 | 0.001 | 119.854 |
| 120054 | 0 | 120.054 |
| 120254 | 0 | 120.254 |
| 120454 | 0 | 120.454 |
| 120654 | 0 | 120.654 |
| 120854 | 0.001 | 120.854 |
| 121054 | 0.00167 | 121.054 |
| 121254 | 0.00167 | 121.254 |
| 121454 | 0.00067 | 121.454 |
| 121654 | 0.001 | 121.654 |
| 121854 | 0.001 | 121.854 |
| 122054 | 0.001 | 122.054 |
| 122254 | 0 | 122.254 |
| 122454 | 0 | 122.454 |
| 122654 | 0 | 122.654 |
| 122854 | 0 | 122.854 |
| 123054 | 0 | 123.054 |
| 123254 | 0 | 123.254 |
| 123454 | 0 | 123.454 |
| 123654 | 0.001 | 123.654 |
| 123854 | 0.001 | 123.854 |
| 124054 | 0.002 | 124.054 |
| 124273 | 0.002 | 124.273 |
| 124473 | 0.002 | 124.473 |
| 124673 | 0.00167 | 124.673 |
| 124873 | 0.00067 | 124.873 |
| 125073 | 0.00067 | 125.073 |
| 125273 | 0 | 125.273 |
| 125473 | 0 | 125.473 |
| 125673 | 0 | 125.673 |
| 125873 | 0 | 125.873 |
| 126073 | 0 | 126.073 |
| 126273 | 0 | 126.273 |
| 126473 | 0 | 126.473 |
| 126673 | 0 | 126.673 |
| 126873 | 0 | 126.873 |
| 127073 | 0 | 127.073 |
| 127273 | 0 | 127.273 |
| 127473 | 0 | 127.473 |
| 127673 | 0 | 127.673 |
| 127873 | 0 | 127.873 |
| 128073 | 0 | 128.073 |
| 128273 | 0 | 128.273 |
| 128473 | 0 | 128.473 |
| 128673 | 0 | 128.673 |
| 128873 | 0.001 | 128.873 |
| 129073 | 0.001 | 129.073 |
| 129273 | 0.001 | 129.273 |
| 129473 | 0 | 129.473 |
| 129673 | 0 | 129.673 |
| 129873 | 0 | 129.873 |
| 130073 | 0 | 130.073 |
| 130273 | 0 | 130.273 |
| 130473 | 0.001 | 130.473 |
| 130673 | 0.001 | 130.673 |
| 130873 | 0.002 | 130.873 |
| 131073 | 0.00167 | 131.073 |
| 131273 | 0.00233 | 131.273 |
| 131473 | 0.002 | 131.473 |
| 131673 | 0.00333 | 131.673 |
| 131873 | 0.00267 | 131.873 |
| 132073 | 0.003 | 132.073 |
| 132273 | 0.001 | 132.273 |
| 132473 | 0.001 | 132.473 |
| 132673 | 0.001 | 132.673 |
| 132873 | 0.001 | 132.873 |
| 133073 | 0.001 | 133.073 |
| 133273 | 0 | 133.273 |
| 133473 | 0 | 133.473 |
| 133673 | 0 | 133.673 |
| 133873 | 0.001 | 133.873 |
| 134073 | 0.001 | 134.073 |
| 134273 | 0.001 | 134.273 |
| 134390 | 0.00924 | 134.39 |
