# Supplementary material for: Towards Improved Molecular Identification Tools in Fine Fescue (Festuca L., Poaceae) Turfgrasses: Nuclear Genome Size, Ploidy, and Chloroplast Genome Sequencing
Source: Front Genet. 2019 Dec 6;10:1223. doi: 10.3389/fgene.2019.01223 (PMC6909427; doi:10.3389/fgene.2019.01223)
Supplement: Supplementary file 7 [file Image_1.pdf]

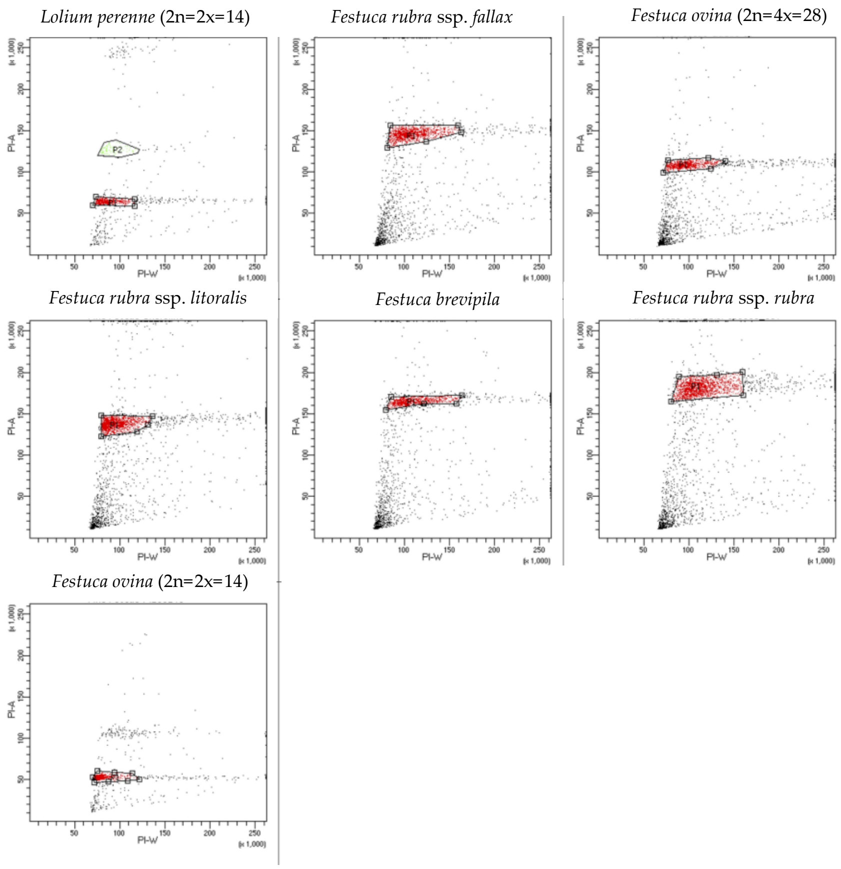

Figure S1: Flow cytometry nuclei population distribution of *L. perenne*, fine fescues, and the diploid USDA PI accession. G1 population for each sample is gated in red, G2 population is gated only in *L. perenne* with green color.
