# Supplementary material for: Towards Improved Molecular Identification Tools in Fine Fescue (Festuca L., Poaceae) Turfgrasses: Nuclear Genome Size, Ploidy, and Chloroplast Genome Sequencing
Source: Front Genet. 2019 Dec 6;10:1223. doi: 10.3389/fgene.2019.01223 (PMC6909427; doi:10.3389/fgene.2019.01223)
Supplement: Supplementary file 8 [file Image_2.pdf]

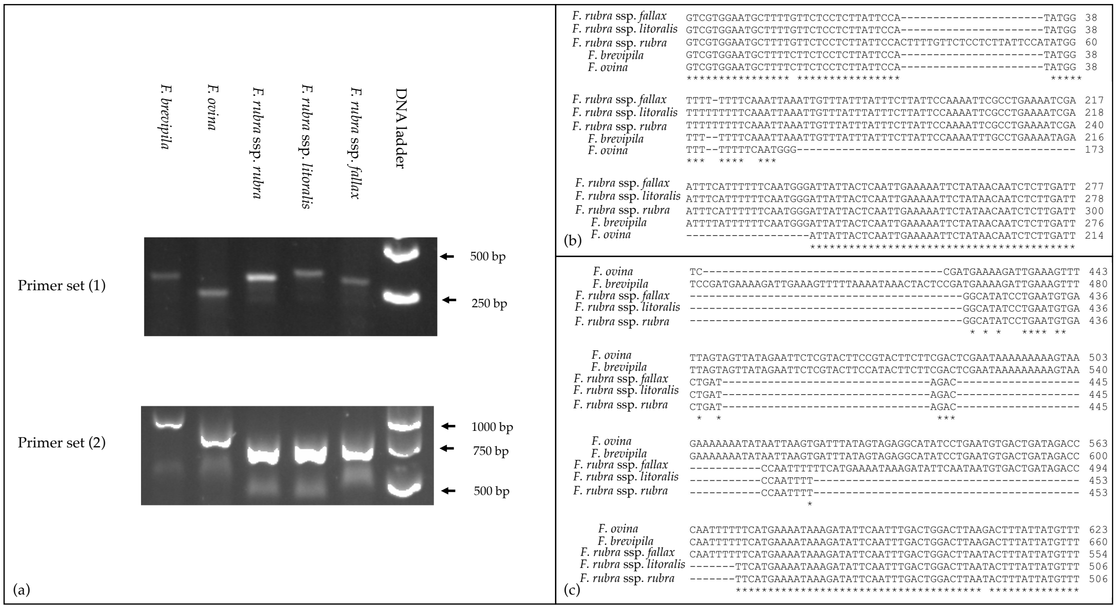

Figure S2. Examples of PCR validation of predicted repeat regions based on fine fescue chloroplast genomes. PCR primers were developed using Primer3 module(Untergasser, Cutcutache et al. 2012). Primers used for the PCR assays (1) Forward primer 5′-GTCGTGGAATGCTTTTGTTCTC-3′; Reverse primer 5′-AGTGGATTCATCAGATGATACA-3′; (2) Forward primer 5′-TTCCTCTTTTCATTG- CAAAGTGGT AT-3′; Reverse primer 5′-TACTCGGAGGTTCGAATCCTTCC-3′. PCR products were examined on 1% agarose gel and gel images showed fragment size separation between different taxa(a). Figure (b) and (c) showed partial sequence alignment of regions amplified by primer sets (1 and 2).
